# Periodontitis and Rheumatoid Arthritis: Shared Pathophysiology, Bidirectional Association, and Therapeutic Implications—A Narrative Review

**DOI:** 10.3390/healthcare14101411

**Published:** 2026-05-20

**Authors:** Neda Najafimakhsoos, Emanuela Pashollari, Nazzarena Malavolta, Francesca Zangari, Claudio Cesari

**Affiliations:** 1 Department of Biomedical and Neuromotor Sciences, University of Bologna, 40125 Bologna, Italy; neda.najafimakhsoos@studio.unibo.it; 2 Department of Dentistry, Vita-Salute San Raffaele University, 20132 Milan, Italy; 3 Casa di Cura Madre Fortunata Toniolo, 40141 Bologna, Italy; 4 Periodontal Unit, Department of Dentistry, Vita-Salute San Raffaele University, 20132 Milan, Italy

**Keywords:** periodontitis, rheumatoid arthritis, systemic inflammation, oral microbiome, *Porphyromonas gingivalis*, autoimmunity, citrullination, anti-citrullinated protein antibodies, microbial dysbiosis, bidirectional association, periodontal therapy, disease-modifying antirheumatic drugs

## Abstract

Periodontitis (PD) and rheumatoid arthritis (RA) are chronic inflammatory disorders that impose substantial individual and societal burdens worldwide. PD is characterized by progressive destruction of the periodontal ligament and alveolar bone, leading to tooth loss, impaired oral function, and sustained systemic inflammatory burden. RA, affecting approximately 0.5–1% of the population, is a chronic autoimmune disease marked by persistent synovial inflammation, progressive joint destruction, disability, and reduced quality of life. Increasing evidence indicates that these conditions are biologically and clinically interconnected. Both diseases share key pathogenic pathways, including microbial dysbiosis, immune dysregulation, chronic inflammation, genetic susceptibility, and aberrant autoantibody responses. Particular attention has focused on keystone periodontal pathogens such as *Porphyromonas gingivalis* and *Aggregatibacter actinomycetemcomitans*, which may promote protein citrullination and the formation of anti-citrullinated protein antibodies (ACPA), thereby providing a plausible mechanistic bridge between periodontal infection and systemic autoimmunity. Shared genetic risk factors, including HLA-DRB1 susceptibility alleles, further support a common host predisposition. Clinical, epidemiological, and translational studies increasingly support a bidirectional association. Individuals with PD appear to have a higher risk of RA development, whereas patients with RA demonstrate greater prevalence, severity, and progression of periodontal disease. Interventional studies suggest that nonsurgical periodontal therapy may reduce local periodontal inflammation, circulating inflammatory biomarkers, and RA disease activity indices, while effective pharmacological control of RA may also improve periodontal outcomes. This narrative review critically evaluates the PD–RA relationship across four interconnected domains: (i) epidemiological and clinical associations between PD and RA, (ii) key mechanisms underlying RA pathogenesis, (iii) shared biological pathways linking both diseases, and (iv) the extent to which treatment of one condition influences the other. Particular emphasis is placed on major sources of heterogeneity and confounding—including smoking, metabolic comorbidities, disease stage, therapeutic exposure, and variable diagnostic definitions—that may explain inconsistencies across the literature. By integrating current mechanistic and clinical evidence, this review provides a structured synthesis that extends beyond a descriptive overview of association studies. A clearer understanding of the periodontal–rheumatologic axis may facilitate risk stratification, identify novel therapeutic targets, and support integrated multidisciplinary care. Targeting both oral and systemic inflammation may improve outcomes in patients with coexisting PD and RA and may potentially reduce the risk or severity of one condition in individuals already affected by the other.

## 1. Introduction

Periodontitis (PD) is a chronic inflammatory disease characterized by progressive destruction of the tooth-supporting apparatus, including the periodontal ligament and alveolar bone, ultimately leading to tooth loss [1]. It represents a major cause of edentulism in adults and contributes substantially to the global public health and economic burden of oral diseases. The worldwide annual cost of oral diseases has been estimated at approximately USD 544 billion, of which PD accounts for nearly USD 79 billion [2].

A growing body of scientific evidence has demonstrated strong associations between PD and several systemic conditions, including cardiovascular disease, diabetes mellitus, respiratory disorders, thyroid dysfunction, and obstructive sleep apnea (OSA) [3]. These associations are largely explained by shared pathophysiological mechanisms such as chronic systemic inflammation, immune dysregulation, and microbial translocation, which may contribute to the development or progression of systemic diseases [4].

**Figure 1:**
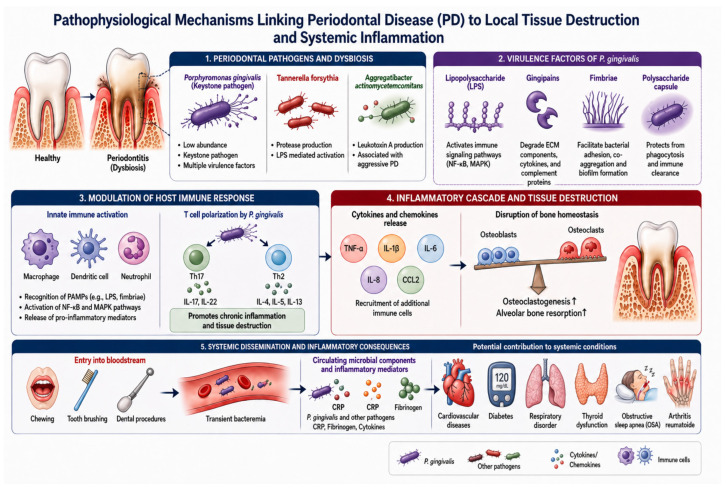
Pathophysiological mechanisms linking periodontal disease to local tissue destruction and systemic inflammation. This schematic illustrates how periodontal dysbiosis and pathogenic biofilm communities, particularly *Porphyromonas gingivalis*, *Tannerella forsythia*, and *Aggregatibacter actinomycetemcomitans*, initiate host immune activation and chronic inflammation. Microbial virulence factors stimulate innate and adaptive immune responses, leading to cytokine and chemokine release, matrix degradation, osteoclastogenesis, connective tissue breakdown, and progressive alveolar bone resorption. In parallel, transient bacteremia and dissemination of microbial products and inflammatory mediators into the systemic circulation may amplify systemic inflammatory burden and potentially contribute to extraoral comorbidities, including cardiovascular, metabolic, respiratory, and rheumatic disorders.

PD results from a complex interaction between pathogenic microorganisms and the host immune response (Figure 1). Among the periodontal pathogens, *Porphyromonas gingivalis* plays a key role in disrupting immune homeostasis and promoting oral microbial dysbiosis [4]. Despite its relatively low abundance, it acts as a keystone pathogen through multiple virulence factors including lipopolysaccharide, gingipains, fimbriae, and a polysaccharide capsule [4]. These factors enable the bacterium to modulate host immune responses, activate inflammatory signaling pathways such as NF κB and MAPK, and evade immune clearance. Gingipains degrade extracellular matrix components, cytokines, and complement proteins, while fimbriae facilitate bacterial adhesion and microbial interactions and the capsule protects the organism from phagocytosis, allowing persistence within periodontal tissues. In addition, *P. gingivalis* alters T cell responses by promoting Th17 and Th2 polarization, thereby sustaining chronic inflammation and periodontal tissue destruction [4].

Other periodontal pathogens further amplify this inflammatory process. *Tannerella forsythia* stimulates immune responses through protease production and lipopolysaccharide-mediated activation of inflammatory pathways, inducing the release of cytokines such as tumor necrosis factor alpha, interleukin 1, and interleukin 6. *Aggregatibacter actinomycetemcomitans*, commonly associated with aggressive forms of PD, produces leukotoxin A which disrupts leukocyte function and enhances macrophage-mediated inflammatory responses [4]. The interaction between periodontal pathogens and host immune cells leads to the release of cytokines and chemokines that amplify local inflammation and recruit additional immune cells. This inflammatory cascade disrupts the balance between osteoblast and osteoclast activity, promoting osteoclastogenesis and progressive alveolar bone resorption [4]. In addition to local tissue destruction, periodontal pathogens and inflammatory mediators such as C-reactive protein and fibrinogen may disseminate into the bloodstream during routine activities including chewing, tooth brushing, or dental procedures, potentially contributing to systemic inflammatory conditions [4].

Rheumatoid arthritis (RA) is a chronic autoimmune inflammatory disease affecting approximately 0.5–1% of the global population, with a higher prevalence in women and a peak onset between 40 and 60 years of age [5,6,7]. It is characterized by synovial inflammation and progressive destruction of cartilage and bone, leading to pain, joint deformity, and functional disability [8,9,10]. The etiology of RA is multifactorial and involves a complex interaction between genetic predisposition, environmental exposures, hormonal influences, and infections, which together contribute to immune dysregulation and the production of autoantibodies [11]. Genetic factors account for approximately 50–60% of disease susceptibility, while environmental triggers, including infections, may act as catalysts in genetically predisposed individuals [12,13]. Clinically, RA manifests as a symmetric inflammatory polyarthritis primarily affecting small joints, with progression to larger joints and systemic involvement in advanced stages [14].

Beyond articular involvement, RA has a substantial impact on overall quality of life. Chronic fatigue, psychological distress, and kinesiophobia-defined as fear of movement due to pain or perceived joint instability-contribute to functional decline, reduced muscle strength, impaired mobility, and limitations in daily activities. These factors frequently lead to increased levels of anxiety and depression, further exacerbating the physical and psychological burden associated with the disease [15].

In addition to its musculoskeletal manifestations, RA is frequently associated with systemic symptoms such as fatigue, low-grade fever, and malaise, and may involve multiple extra articular organ systems including the cardiovascular, pulmonary, cutaneous, renal, gastrointestinal, ocular, neurological, and skeletal systems [16,17,18]. The presence of these systemic manifestations further highlights the complex multisystem nature of the disease.

Increasing evidence suggests a bidirectional relationship between PD and RA, indicating that these two chronic inflammatory diseases may influence each other through shared pathogenic pathways. A growing body of literature supports overlapping mechanisms involving microbial dysbiosis, immune dysregulation, and systemic inflammatory mediators, which may contribute to both the initiation and progression of these conditions. However, the strength and consistency of this association remain variable, and the underlying mechanisms are not yet fully understood.

Accordingly, the aim of this narrative review is to critically evaluate the bidirectional relationship between PD and RA, structured across four interconnected domains: (i) the association between PD and RA, (ii) the pathogenesis of rheumatoid arthritis, (iii) shared biological links between the two conditions, and (iv) the influence of one condition’s treatment on the other. In addition, special attention is given to sources of heterogeneity and potential confounding factors that may explain inconsistencies across studies. By integrating these aspects, this review moves beyond a descriptive overview to provide a structured synthesis of the biological and clinical interplay between PD and RA. A clearer understanding of these interactions may support the development of integrated therapeutic strategies and foster closer collaboration between dental and medical disciplines in the management of affected patients.

The manuscript is organized according to these four interconnected domains to ensure a structured and critical synthesis of the available evidence.

## 2. Materials and Methods

This narrative literature review was conducted to evaluate the bidirectional relationship between RA and PD, with a focus on epidemiological associations, shared pathogenic mechanisms, and potential therapeutic interactions.

A structured literature search was performed in PubMed/MEDLINE and Web of Science databases to enhance transparency and methodological clarity. The search included articles published between January 2000 and March 2026. The search strategy combined Medical Subject Headings (MeSH) and free-text terms, including “periodontal disease,” “periodontitis,” “rheumatoid arthritis,” “association,” “treatment,” “pathophysiology,” and “molecular pathways,” using Boolean operators (AND/OR).

In accordance with the narrative design, the objective of the search was not to identify all available studies, but to retrieve representative, high-quality, and conceptually relevant evidence that meaningfully contributes to understanding the relationship between these conditions.

Study selection was conducted in two stages. First, titles and abstracts were screened for relevance. Subsequently, full texts of potentially eligible studies were assessed. The selection process was performed by two independent reviewers, and any disagreements were resolved through discussion with another reviewer. Study inclusion was guided by scientific relevance, methodological robustness, and contribution to the predefined thematic framework, rather than exhaustive retrieval.

Studies were considered eligible if they contributed to understanding the association between PD and RA, including epidemiological evidence, shared biological or immunological mechanisms, and the impact of therapeutic interventions on either condition.

Inclusion criteria comprised human clinical studies (cross-sectional, case–control, cohort studies, and clinical trials), as well as systematic reviews and meta-analyses addressing the relationship between RA and PD. Studies investigating shared pathogenic pathways or evaluating the effects of periodontal or rheumatologic treatments were also included. Only articles published in English were considered.

Exclusion criteria included animal studies, case reports and small case series with limited methodological robustness, studies not directly relevant to the scope of this review, and non-English publications.

Data synthesis was qualitative due to heterogeneity in study designs, populations, and outcome measures. Findings were synthesized narratively and organized into thematic sections to enhance clarity, coherence, and clinical interpretability. Key studies are also summarized in tables at the end of each section to provide a structured overview of the evidence.

The results are presented in structured sections, including epidemiological evidence, underlying pathogenic mechanisms, and therapeutic interactions. The potential implications for interdisciplinary management are also discussed.

### Methodological Considerations and Limitations

This review was conducted as a narrative synthesis and does not follow a formal systematic review protocol (e.g., PRISMA). Consequently, no standardized risk of bias assessment tool was applied. Although efforts were made to include studies of high methodological quality and clinical relevance, the process inherently involves selective interpretation and may be subject to selection bias, publication bias, and omission of some relevant studies. These limitations are acknowledged as intrinsic to the narrative review methodology.

## 3. Results and Discussion

### 3.1. Bidirectional Association Between Periodontitis and Rheumatoid Arthritis

#### 3.1.1. Evidence Linking Periodontitis to Rheumatoid Arthritis

Several clinical investigations have evaluated whether PD is associated with RA (Table 1).

Chou et al. [19] reported that PD is independently associated with an increased risk of RA in a dose-dependent manner, with risk increasing according to periodontal disease severity and treatment intensity. This association remained significant after adjustment for age, sex, and diabetes history. Periodontal exposure was defined as having at least one ambulatory visit with a diagnosis of PD, together with evidence of periodontal treatment, antibiotic therapy, or repeated dental scaling (up to three times). PD severity was additionally assessed using healthcare utilization metrics, including number of visits, treatment costs, antibiotic prescriptions, and periodontal surgical procedures. RA was defined as at least one ambulatory visit with a confirmed diagnosis followed by issuance of a catastrophic illness certificate. Age, sex, and diabetes were included as confounding variables. Treated diabetes was associated with a lower risk of RA development, with a stronger protective effect observed in men. However, no statistically significant interaction between sex and diabetes on RA risk was reported.

A systematic review reported that PD is associated with an increased risk RA, supporting its role as a potential risk factor for the disease. The association appeared to be influenced by disease duration, with a stronger effect observed in patients with longer-standing RA compared to those with shorter disease duration. PD was also more strongly associated with incident RA than with mixed or prevalent RA populations, suggesting a greater impact on disease onset. However, the study did not account for differences in follow-up duration across included studies, nor did it adequately adjust for variability in the time interval between case and control assessments, which may have introduced methodological heterogeneity and potential bias in the pooled estimates [20].

Another systematic review comparing patients diagnosed with RA for less than three years showed that the proportion of ACPA was higher when PD was present. In contrast, studies involving RA patients diagnosed for more than three years demonstrated a similar frequency of ACPA regardless of periodontal status. Overall, serum ACPA levels were higher in RA patients with PD compared to those without PD. In addition, PD was associated with increased seropositivity for ACPA and rheumatoid factor (RF), with higher circulating levels observed in RA patients with concomitant periodontal disease, suggesting a potential role of periodontal inflammation in systemic immune modulation [21].

However, the literature is not entirely consistent. A cross-sectional study found no evidence of an association between PD and RA. PD was defined as ≥2 non-adjacent interdental sites with clinical attachment loss (CAL) and probing depth ≥ 4 mm with bleeding on probing (BOP). RA was diagnosed based on ACR criteria with DAS28-CRP ≥ 3.2. Patients with RA exhibited less severe periodontal destruction compared to those without RA. Periodontal clinical parameters showed an inverse relationship with key RA biomarkers, suggesting that increased periodontal severity does not parallel systemic inflammatory burden in RA. Importantly, common confounding factors, including age, smoking, diabetes, osteoporosis, and *P. gingivalis*, did not significantly influence this relationship. The lower periodontal destruction observed among patients with RA may be explained by the potential modifying effect of long-term anti-rheumatic therapy, such as DMARDs and corticosteroids, on periodontal status by reducing inflammation, which may partially account for the lack of association [22].

A possible association between PD and an increased risk of RA has been reported in multiple studies, although findings are not fully consistent. Overall, the majority of evidence supports a positive association between PD and RA; however, substantial heterogeneity exists, and the observed relationship should be interpreted within a broader systemic inflammatory and immunological context.

#### 3.1.2. Evidence Linking Rheumatoid Arthritis to Periodontitis

Several clinical investigations have evaluated whether RA is associated with PD (Table 2).

This nationwide registry-based study provides strong epidemiological evidence of a consistent association between RA and an increased occurrence of PD. The relationship is biologically plausible and clinically relevant, as both conditions share overlapping inflammatory and immunological pathways. The association is not uniform across patients and is significantly influenced by disease activity, with higher RA activity associated with greater periodontal burden. Temporal modeling further strengthens the findings by ensuring that periodontal outcomes occur after RA onset, supporting a directional relationship. Moreover, the stronger association observed in newly diagnosed patients suggests that early disease phases may play an important role in systemic inflammatory interactions. The study also reports that the risk of PD in patients with RA was highest among those with ten or more rheumatology visits, suggesting a relationship with disease severity or healthcare utilization as a proxy of activity. Moreover, the risk of PD increased with a higher number of comorbid conditions, including diabetes mellitus and myocardial infarction, indicating that systemic burden may further amplify susceptibility to periodontal disease. Despite these strengths, the interpretation of the findings should be made with appropriate caution. The use of administrative diagnostic codes as proxies for clinical conditions introduces a risk of misclassification for both RA and PD. The analyses appropriately adjusted for several key confounders, including sex, age, diabetes mellitus, and myocardial infarction, and further employed multivariable Cox regression models alongside inverse probability treatment weighting to account for prior PD history and reduce bias related to disease chronology. Nevertheless, residual confounding is likely to persist, particularly due to the absence of information on smoking, which represents a major shared risk factor for both conditions. Additional unmeasured variables, including socioeconomic status, oral hygiene practices, and medication use, were not available and may have influenced the observed associations. Overall, while this study applies strong methodological approaches and provides compelling evidence supporting an association between RA and PD, causal inference remains limited. Further studies incorporating detailed clinical, behavioral, and lifestyle data are required to better clarify the underlying mechanisms and strengthen causal interpretation [23].

This cross-sectional study demonstrates that patients with RA have a significantly higher prevalence of PD compared with systemically healthy individuals, suggesting that RA-related immune dysregulation increases susceptibility to periodontal tissue breakdown. Markers of disease severity, including longer disease duration and older age, were positively associated with PD, supporting a dose response relationship between systemic inflammatory burden and periodontal destruction and strengthening the biological plausibility of a bidirectional link between the two conditions. From a mechanistic perspective, the findings support a shared cytokine-mediated inflammatory pathway. Higher levels of TNF-α and IL-1 beta in gingival crevicular fluid (GCF) were observed in patients with RA and PD, indicating amplification of local periodontal inflammation in the presence of systemic autoimmune activity. In addition, serum CRP and local TNF-α were identified as independent predictors of IL-1 beta expression, suggesting a systemic to local inflammatory amplification loop. At the same time, periodontal inflammation may further contribute to systemic immune activation through sustained cytokine release and bacterial antigen dissemination, potentially worsening RA activity. The study attempted to reduce major confounding by excluding patients with diabetes mellitus, smoking history, use of immunosuppressive or antibiotic therapy, and other autoimmune diseases. However, important limitations remain, including the cross-sectional design which prevents assessment of temporality, lack of detailed oral hygiene assessment, absence of socioeconomic and dietary data, and no stratification of RA medications or disease severity, leaving the possibility of residual confounding [24]. The study also reported that longer duration of morning stiffness was associated with higher concentrations of periodontal disease index, accompanied by increased probing depth, as well as elevated levels of IL-1 beta and TNF-α in GCF [24].

This study excluded patients with fewer than 15 teeth and reported that the prevalence of moderate and severe PD was significantly higher in patients with RA compared with non-RA controls. BOP and gingival index (GI) were positively correlated with RA disease duration, whereas probing pocket depth and CAL showed no such correlation. Early RA, defined as disease duration of less than one year, did not show significant differences in periodontal involvement compared with other groups, while longstanding disease with duration greater than ten years was associated with significantly higher GI and BOP. In addition, probing pocket depth was significantly lower in patients carrying the HLA-DRB1 shared epitope compared with those without it. This study included patients with diabetes, which may represent a potential confounding factor [25].

This cross-sectional study by González et al. [26] evaluated the association between RA characteristics and periodontal disease severity in a clinically defined RA population. Greater periodontal severity was observed in patients with longer disease duration, higher disease activity, and the presence of rheumatic nodules, with disease activity and nodules showing the strongest associations. Patients with disease duration of sixteen years or more had approximately four times higher likelihood of severe PD compared with those with shorter duration, whereas no significant association was found with corticosteroid therapy or disease duration when considered independently. Conventional inflammatory markers and pharmacologic treatments were not significantly related to periodontal outcomes, suggesting that clinical phenotype may be more relevant than isolated laboratory measures. Strict exclusion criteria, including smoking, diabetes mellitus, other autoimmune diseases, secondary Sjogren syndrome, recent periodontal or antibiotic therapy, and having fewer than six teeth, improved internal validity. However, residual confounding remains due to lack of data on oral hygiene, dental care access, and socioeconomic status, as well as potential effects of impaired manual dexterity in RA. All patients were receiving medications such as methotrexate, corticosteroids, and nonsteroidal anti-inflammatory drugs, which may have reduced inflammation and masked periodontal severity. Additional limitations include the cross-sectional design, small sample size, and selection bias due to inclusion of only patients with both RA and PD without a RA control group.

This pilot cross-sectional study reported that PD was more prevalent and more severe in patients with RA compared with those with osteoarthritis, and that patients with RA were more likely to have PD. After adjustment for smoking and diabetes mellitus, moderate to severe PD remained significantly associated with RA, with an approximately twofold increase in odds for each incremental increase in periodontal disease severity. No association was observed between periodontal severity and RA disease duration or medication use, including glucocorticoids and disease modifying antirheumatic drugs (DMADs). However, seropositive patients, including those positive for RF and anti-citrullinated protein antibodies, were more likely to present with moderate to severe PD, whereas no associations were found with other clinical measures of disease activity or severity. Patients with fewer than four teeth were excluded. These findings suggest that the autoimmune profile, rather than clinical disease activity, may play a more important role in periodontal involvement, supporting shared immunoinflammatory mechanisms related to protein citrullination and immune dysregulation. However, major residual confounding remains, particularly due to the absence of oral hygiene assessment, which is critical as impaired manual dexterity in RA may worsen plaque control and contribute to PD. Additional limitations include the cross-sectional design, small sample size, and pilot nature of the study. Overall, the study provides preliminary evidence supporting an association between RA and PD, particularly emphasizing the role of serological markers, although the findings should be interpreted with caution due to methodological constraints [27].

Patients with RA, including both early and chronic forms, demonstrate a significantly higher prevalence of moderate PD and overall poorer periodontal status compared with systemically healthy controls, and this impairment persists despite treatment with synthetic and biological DMADs. The prevalence of *Porphyromonas gingivalis* is increased in early untreated disease with probing depth ≥ 4 mm compared with chronic disease and controls, and antirheumatic therapy does not significantly influence these findings. This well-designed prospective study provides strong longitudinal evidence supporting an independent association between RA and PD across the full disease spectrum. Standardized definitions based on CDC and AAP criteria for PD and validated clinical criteria for RA enhance internal validity. The findings indicate that periodontal destruction is not substantially reversed by systemic anti-inflammatory treatment, suggesting partially independent pathogenic mechanisms. The increased presence of key periodontal pathogens supports a biologically plausible link through microbial driven immune dysregulation, although persistent periodontal damage appears to progress independently once established. Major confounders including smoking, age, sex, and oral hygiene were accounted for and did not materially alter the association, although residual confounding related to socioeconomic status, access to dental care, comorbidities such as diabetes and cardiovascular disease, and behavioral factors including impaired oral hygiene practices in RA patients may contribute. The predominantly female tertiary care population may limit generalizability, and single-time point assessment of controls restricts temporal comparison. Overall, the study supports a robust independent association between RA and increased periodontal disease burden, highlighting the need for integrated medical and periodontal management [28].

This case–control study investigates whether PD modifies the relationship between RA and cardiovascular risk by evaluating atherogenic lipid indices rather than treating periodontal variables as isolated endpoints. The population consists of treated RA patients and clinically comparable controls, which strengthens internal validity but introduces treatment-related confounding. The study demonstrates that periodontal inflammation and severity are consistently associated with an adverse lipid profile characterized by increased low density lipoprotein cholesterol, reduced high density lipoprotein cholesterol, and elevated atherogenic indices. The presence of a statistically significant interaction between periodontal inflammation and RA for LDL cholesterol suggests a disease-specific amplification effect, supporting a synergistic rather than independent association. A critical confounder is pharmacologic therapy, as all RA patients were receiving corticosteroids, conventional disease modifying drugs, and biologic agents, which are known to influence both systemic inflammation and lipid metabolism. This may attenuate or distort the observed associations. Additional confounders include smoking, age, sex, and metabolic conditions such as diabetes, obesity, and hypertension, which are either partially controlled or not fully explored despite their strong biological relevance to both PD and cardiovascular risk. The population is relatively small and derived from a single clinical center, limiting statistical power and generalizability. The cross-sectional nature of the analysis prevents causal inference, and the absence of reported effect sizes and confidence intervals limits the interpretation of the strength and precision of associations. Furthermore, exclusion of major systemic diseases improves internal validity but reduces applicability to real world multimorbid populations where these interactions are most relevant. Overall, the study supports a biologically plausible model in which PD acts as a modifying factor that exacerbates the atherogenic burden in RA through shared inflammatory and metabolic pathways, rather than serving as a primary independent determinant of cardiovascular risk [29].

This population based retrospective cohort study using Taiwan National Health Insurance Research Database shows that patients with RA have higher prevalence and frequency of dental visits and increased incidence of dental disorders including dental caries, pulpitis, gingivitis, PD and oral ulcer compared with matched individuals without RA. RA diagnosis is based on catastrophic illness certification requiring specialist verification according to established criteria, while dental outcomes are identified using ICD 9 CM codes confirmed by dentists. The study includes adjustment for multiple comorbidities including hypertension, diabetes mellitus, cardiovascular disease, chronic pulmonary disease, malignancy, dyslipidemia, cerebrovascular disease, musculoskeletal disorders, peptic ulcer disease, chronic kidney disease, and liver disease, and excludes patients with Sjogren syndrome. Patients with RA show higher prevalence of chronic pulmonary disease, musculoskeletal disorders, and peptic ulcer disease and lower prevalence of cancer compared with controls. Limitations include use of administrative data, lack of clinical parameters for periodontal disease, absence of information on disease severity and duration, and lack of data on behavioral factors such as smoking and oral hygiene. The findings indicate increased dental disease burden and healthcare utilization in patients with RA [30].

In this pilot case–control study, salivary flow cytometry revealed significantly elevated CD11b and CD38 expression in patients with RA compared with controls, supporting the presence of systemic immune activation detectable in oral fluids. Importantly, CD11b demonstrated consistent and significant associations with key periodontal inflammatory parameters, including BOP, plaque accumulation, and periodontal severity index, suggesting that this integrin may reflect the intensity of local periodontal inflammation in the context of systemic autoimmunity. Mechanistically, CD11b is known to regulate monocyte adhesion, macrophage activation, and osteoclast differentiation through tyrosine kinase–dependent pathways and downstream activation of NFATc1 and c-Fos signaling. These pathways converge on RANKL-mediated osteoclastogenesis, providing a plausible biological explanation for the observed association between CD11b expression and periodontal tissue destruction. The concomitant elevation of CD38 further supports a state of immune cell activation in RA patients, although its lack of consistent correlation with periodontal parameters suggests a more systemic rather than locally driven role. The bidirectional nature of the RA–PD relationship is further reinforced by the correlation between periodontal inflammation and established autoimmune markers such as anti-citrullinated peptide antibodies and RF. These findings align with the hypothesis that dysbiotic oral microbiota may contribute to systemic autoimmunity through citrullination pathways, while systemic inflammatory priming in RA may exacerbate periodontal breakdown through enhanced osteoclastogenic activity. However, the study has limitations that must be considered when interpreting causality. The small sample size for flow cytometry analysis limits statistical power and generalizability. In addition, the use of immunomodulatory therapy in RA patients may have attenuated CD11b expression, suggesting that the observed differences may underestimate the true biological effect. Finally, the cross-sectional design precludes inference of temporal or causal relationships between immune activation and periodontal destruction.

Despite these limitations, the use of saliva-based flow cytometry represents a novel and minimally invasive approach for assessing immune–periodontal interactions. Overall, CD11b emerges as a promising candidate biomarker linking systemic autoimmune inflammation and periodontal disease activity, supporting the concept of a shared immuno-osteoclastogenic axis between RA and PD [31].

This age and gender matched cross-sectional case–control study shows that patients with RA have significantly higher periodontal attachment loss compared with controls. PD is defined as CAL greater than 4 mm measured by standardized periodontal examination, while RA is diagnosed using established classification criteria based on clinical and laboratory evaluation ensuring diagnostic validity. The association remains significant after adjustment for age, gender, smoking, alcohol consumption, body mass index, and education, with only age and RA retained as independent predictors. Oral hygiene measures including plaque index and GI explain only a small proportion of the association, indicating limited mediation by hygiene status. The study population includes patients with established RA receiving DMADs, corticosteroids, and biologic therapies, with additional systemic conditions such as osteoporosis, coronary heart disease, diabetes mellitus, hypertension, and dyslipidemia considered as potential confounders. Limitations include cross-sectional design preventing causal inference, small sample size limiting statistical power and confounder adjustment, potential selection bias from clinic based controls, and residual confounding from medication and lifestyle factors. Overall, the findings indicate an independent association between RA and periodontal destruction with only partial explanation by oral hygiene [32].

Nevertheless, some studies have produced conflicting findings.

This nationally representative cross-sectional study using Korean National Health and Nutrition Examination Survey data evaluates the relationship between RA and oral health outcomes using standardized periodontal examination based on the Community Periodontal Index and clinical assessment of tooth loss. PD was defined using objective probing depth criteria and RA was defined as physician diagnosed disease with current treatment. After full multivariable adjustment there was no statistically significant independent association between RA and PD, indicating that crude associations are largely explained by shared systemic and behavioral risk factors rather than a direct relationship. In contrast, RA was associated with increased tooth loss in younger individuals, while no association was observed in older adults, suggesting an age dependent effect where systemic disease burden may contribute more to early cumulative dental deterioration but becomes less detectable with advancing age due to dominant background tooth loss and long term exposure to confounders. Major confounders include smoking, diabetes mellitus, socioeconomic status, oral hygiene behavior, and body mass index, all of which influence both RA and periodontal health. Additional potential confounding arises from disease severity, duration, and pharmacologic treatment including immunosuppressive therapy, as well as functional limitation in RA that may impair oral hygiene practices and indirectly contribute to tooth loss. Limitations include the cross-sectional design which prevents causal inference, reliance on self-reported RA without serological confirmation or formal classification criteria, and residual confounding from unmeasured systemic inflammation and behavioral factors. Periodontal assessment based on partial recording may also underestimate true disease burden. Overall, the findings suggest that RA is not independently associated with PD but may contribute to earlier tooth loss in younger individuals through a combination of systemic inflammation, functional impairment, and shared metabolic and behavioral risk factors rather than a direct periodontal-specific causal mechanism [33].

This matched cross-sectional study in an Indonesian population shows that RA is not associated with increased prevalence or overall severity of PD when assessed using full-mouth clinical examination and multiple definitions of disease. PD prevalence was defined using established case definitions, and severity was evaluated through probing depth, CAL, BOP, and additional measures including periodontal epithelial surface area and inflamed surface area. RA diagnosis was established by rheumatologists according to validated classification criteria. No differences were observed in prevalence or in nearly all clinical measures of periodontal severity between patients with RA and matched controls. However, patients with RA exhibited a reduced periodontal epithelial surface area, indicating a lower amount of healthy periodontal tissue. In addition, a tendency toward higher systemic inflammatory markers was observed in patients with RA and severe PD. A major confounder is pharmacologic treatment, as all patients with RA were receiving anti-inflammatory and antirheumatic drugs, while controls were not, which may have attenuated periodontal inflammation. Smoking and socioeconomic status were controlled through matching, while body mass index differed between groups and may have influenced systemic inflammation. Impaired oral hygiene related to functional limitations was considered but not supported by plaque findings. The population is geographically and ethnically specific with a high background prevalence of PD, which may reduce the ability to detect differences. Exclusion of systemic diseases such as diabetes improves internal validity but limits generalizability. Limitations include the cross-sectional design, reliance on matched and univariate analyses, potential residual confounding, and the influence of pharmacologic treatment. Overall, the findings indicate no observed association between RA and PD prevalence or severity, although differences in periodontal tissue characteristics and trends in systemic inflammation suggest a possible interaction that warrants further investigation [34].

#### 3.1.3. Integrated Interpretation of the Bidirectional Association Between Periodontitis and Rheumatoid Arthritis

Overall, the available body of evidence supports a bidirectional association between PD and RA; however, the strength, consistency, and clinical interpretation of this relationship remain influenced by substantial heterogeneity across studies. This heterogeneity reflects important methodological and clinical differences, including notable variations in the definitions and diagnostic criteria of both conditions, ranging from early symptom-based classification of RA to confirmed established disease, as well as differences in periodontal case definitions and severity grading. In addition, variability in RA stage, disease activity, and disease duration influences systemic inflammatory burden and periodontal outcomes, further limiting direct comparability and contributing to inconsistent findings.

Importantly, multiple confounding factors must be carefully considered when interpreting this association. Shared risk factors such as smoking, diabetes, oral hygiene practices, and socioeconomic status may independently influence both periodontal and rheumatologic outcomes. Furthermore, the widespread use of immunosuppressive therapies in RA may attenuate clinical signs of periodontal inflammation, potentially leading to underestimation of disease prevalence and severity, while increased healthcare utilization in these patients may introduce detection bias. The presence of overlapping autoimmune conditions, particularly secondary Sjögren-like syndrome, may further modify periodontal status through xerostomia and consequent alterations in the oral microbiome, suggesting that part of the observed association may be mediated by salivary dysfunction rather than a direct pathogenic link. Patients with RA are frequently immunosuppressed and undergo closer medical surveillance, which may increase the likelihood of detecting periodontal disease compared with the general population.

Beyond RA, emerging genetic and epidemiological evidence suggests that the relationship between PD and immune-mediated inflammatory disorders (IMIDs) may extend beyond RA, supporting a broader shared immuno-inflammatory susceptibility. A bidirectional Mendelian randomization analysis has shown that systemic lupus erythematosus and Sjögren’s syndrome may causally increase the risk of PD, while PD may also influence the risk of selected IMIDs, including systemic lupus erythematosus. These findings support the existence of shared genetic and immune regulatory pathways across systemic autoimmune and endocrine conditions, including hypothyroidism, suggesting that periodontal disease may represent a clinical manifestation of a broader systemic immune dysregulation phenotype rather than a disease-specific association limited to RA [35]. These findings suggest that periodontal disease may represent a manifestation of systemic immune dysregulation within a wider network of immune-mediated inflammatory disorders.

Rather than being restricted to a single autoimmune entity, PD appears to reflect a systemic immune dysregulation state within a wider network of IMIDs.

Citrullinated proteins generated within the periodontal environment can disseminate systemically, promoting autoantibody production and contributing to inflammatory processes in distant tissues, including the joints. Experimental and clinical evidence shows that infection with *Porphyromonas gingivalis* or *Aggregatibacter actinomycetemcomitans* is associated with increased systemic inflammation, elevated cytokines such as interleukin 6, and more severe arthritis phenotypes [36]. Together, these findings support the concept that bacterially driven citrullination represents a key mechanistic bridge between PD and RA, highlighting its relevance as a potential therapeutic target [36].

Peptidylarginine deiminase 4 (PAD4)-mediated citrullination drives chromatin decondensation during neutrophil extracellular trap (NET) formation, thereby exacerbating myocardial ischemia–reperfusion injury. This process disrupts endothelial integrity, increases vascular permeability, and promotes thrombogenesis through interactions with von Willebrand factor, leading to microvascular obstruction, delayed tissue repair, and sustained inflammation, ultimately worsening cardiac function. Given that *P. gingivalis*, through its bacterial peptidylarginine deiminase (PPAD), directly citrullinates histone H3, and *A. actinomycetemcomitans*, through leukotoxin A, induces hyperactivation of host PAD4, these pathobionts may synergistically contribute to cardiovascular pathology via NET-dependent mechanisms [36]. Furthermore, the coexistence of RA and PD is synergistically associated with a more atherogenic profile, highlighting periodontal health as an important modifiable factor in reducing cardiovascular risk in patients with RA [29].

Collectively, these methodological and clinical variations may partially account for the inconsistencies observed across studies and underscore the need for standardized diagnostic criteria and rigorous adjustment for confounding variables. Importantly, they also raise the question of whether the observed association reflects a true shared pathogenic mechanism or is at least partly driven by systemic inflammation, treatment effects, and overlapping clinical conditions.

From a mechanistic perspective, the most consistent and biologically plausible pathway linking these conditions involves bacterially driven citrullination and subsequent immune activation. Periodontal pathogens capable of inducing protein citrullination, together with dysregulated neutrophil responses and enhanced formation of citrullinated antigens, provide a coherent framework connecting local periodontal infection with systemic autoimmunity. Although additional mechanisms such as systemic dissemination of inflammatory mediators have been proposed, current evidence most strongly supports the central role of citrullination-related pathways in mediating this interaction.

Finally, discrepancies across studies likely reflect substantial methodological variability. Differences in study design, population characteristics, smoking exposure, RA severity, therapeutic regimens, and periodontal diagnostic criteria all contribute to inconsistent findings. Inadequate adjustment for key confounders further limits comparability across studies. These methodological differences likely explain much of the heterogeneity observed in the literature and highlight the need for standardized definitions and rigorously controlled prospective studies in future research.

Taken together, these findings indicate that the association between PD and RA is multifactorial and shaped by a complex interplay of shared inflammatory pathways, systemic conditions, confounding factors, and methodological variability. This integrated perspective supports the concept of a bidirectional axis embedded within a broader systemic inflammatory network, rather than a simple direct causal relationship, and underscores the need for standardized diagnostic criteria and well-controlled prospective studies to further clarify these interactions. Importantly, the available evidence further suggests that this association is more likely to reflect a shared systemic inflammatory and immunological susceptibility rather than a disease-specific interaction, thereby supporting the concept of a broader immune-mediated inflammatory phenotype.

### 3.2. Pathogenesis of Rheumatoid Arthritis

**Figure 2:**
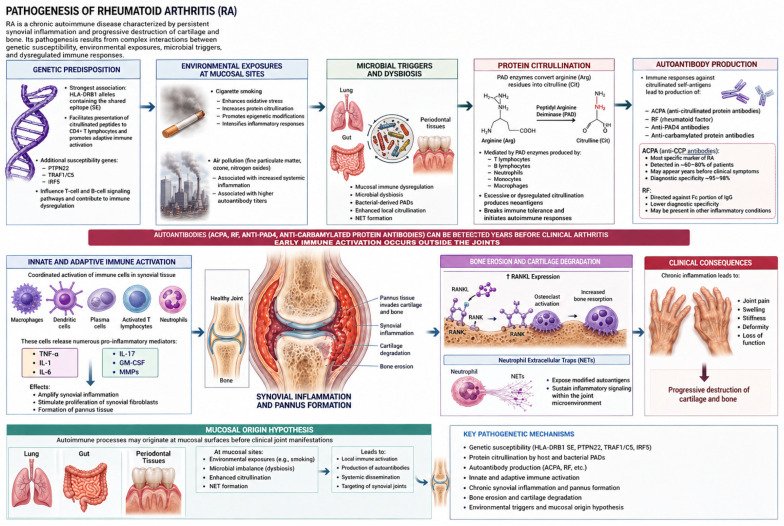
Pathogenesis of rheumatoid arthritis: integrated mechanisms underlying autoimmunity, synovial inflammation, and joint destruction. This schematic summarizes the multifactorial pathogenesis of rheumatoid arthritis (RA), highlighting interactions among genetic susceptibility, environmental exposures, and mucosal microbial triggers that promote protein citrullination and loss of immune tolerance. Subsequent autoantibody production, including anti-citrullinated protein antibodies (ACPA) and rheumatoid factor (RF), contributes to systemic immune activation and chronic synovial inflammation. Persistent cytokine signaling, pannus formation, osteoclastogenesis, and neutrophil extracellular trap (NET) release drive cartilage degradation, bone erosion, and progressive joint dysfunction. The figure also emphasizes the mucosal origin hypothesis, whereby autoimmune processes may begin in the lung, gut, or periodontal tissues before the onset of clinical arthritis.

RA is a chronic autoimmune disease characterized by persistent synovial inflammation and progressive destruction of cartilage and bone. Its pathogenesis results from complex interactions between genetic susceptibility, environmental exposures, microbial triggers, and dysregulated immune responses [37] (Figure 2).

Genetic predisposition plays a central role in disease development. The strongest genetic association involves specific HLA-DRB1 alleles containing the shared epitope, which facilitate the presentation of citrullinated peptides to CD4^+^ T lymphocytes and promote adaptive immune activation. Additional susceptibility genes, including *PTPN22*, *TRAF1/C5*, and *IRF5*, further influence T-cell and B-cell signaling pathways and contribute to immune dysregulation [38].

A fundamental molecular mechanism in RA is protein citrullination, a post-translational modification in which peptidyl arginine deiminase (PAD) enzymes convert arginine residues into citrulline. While citrullination normally participates in physiological cellular regulation, excessive or dysregulated activity produces neoantigens that can break immune tolerance and initiate autoimmune responses [39,40]. Citrullination is mediated by PAD enzymes produced by immune cells, including T lymphocytes, B lymphocytes, neutrophils, monocytes, and macrophages, leading to the production of ACPA [41]. This post-translational modification alters protein structure and, in genetically susceptible individuals such as those carrying shared epitope alleles, may induce immune responses against citrullinated self-antigens [41].

This process results in the formation of ACPA, which represent one of the most specific immunological markers of RA. These antibodies are detectable in approximately 60–80% of patients and may appear years before the onset of clinical symptoms, with diagnostic specificity approaching 95–98% when detected through anti-cyclic citrullinated peptide (anti-CCP) assays [42,43,44]. In contrast, RF, an antibody directed against the Fc portion of immunoglobulin G, demonstrates lower diagnostic specificity and may be present in other inflammatory conditions [45]. Citrullination associated with host-derived PAD enzymes may be augmented by bacterial-derived PADs, enhancing the production of ACPA that can precede the development of RA and therefore play an etiological role in its pathogenesis [11].

The inflammatory cascade in RA is maintained through coordinated activation of innate and adaptive immune responses. Synovial tissues become infiltrated with macrophages, dendritic cells, plasma cells, and activated T lymphocytes that release numerous pro-inflammatory mediators, including tumor necrosis factor-α, interleukin-1, interleukin-6, interleukin-17, granulocyte–macrophage colony-stimulating factor (GM-CSF), and matrix metalloproteinases. These mediators amplify synovial inflammation and stimulate proliferation of synovial fibroblasts, resulting in the formation of pannus tissue that invades cartilage and bone. Increased expression of receptor activator of nuclear factor kappa B ligand promotes osteoclast differentiation and accelerates bone erosion and cartilage degradation [46]. Neutrophil extracellular traps further intensify autoimmune activation by exposing modified autoantigens and sustaining inflammatory signaling within the joint microenvironment [23].

Accumulating evidence suggests that autoimmune processes in RA may originate at mucosal surfaces before clinical joint manifestations appear. According to the mucosal origin hypothesis, immune dysregulation and microbial dysbiosis at mucosal sites, such as the lung, gastrointestinal tract, and periodontal tissues, may initiate autoimmune responses that subsequently disseminate systemically to synovial joints. Autoantibodies, including ACPA, RF, anti-PAD4 antibodies, and anti-carbamylated protein antibodies, can be detected years before the development of clinical arthritis, suggesting that early immune activation occurs outside the joints [11,47].

Environmental exposures at mucosal sites, particularly cigarette smoking and microbial imbalance, enhance local citrullination processes and promote neutrophil extracellular trap formation, facilitating the early stages of rheumatoid arthritis-related autoimmunity. Cigarette smoking represents one of the strongest risk factors for RA because it enhances oxidative stress, increases protein citrullination, and promotes epigenetic modifications that intensify inflammatory responses. Air pollution and exposure to fine particulate matter, ozone, and nitrogen oxides have also been associated with increased systemic inflammation and higher autoantibody titers [18,48,49].

### 3.3. Biological Link Between Periodontitis and Rheumatoid Arthritis

**Figure 3:**
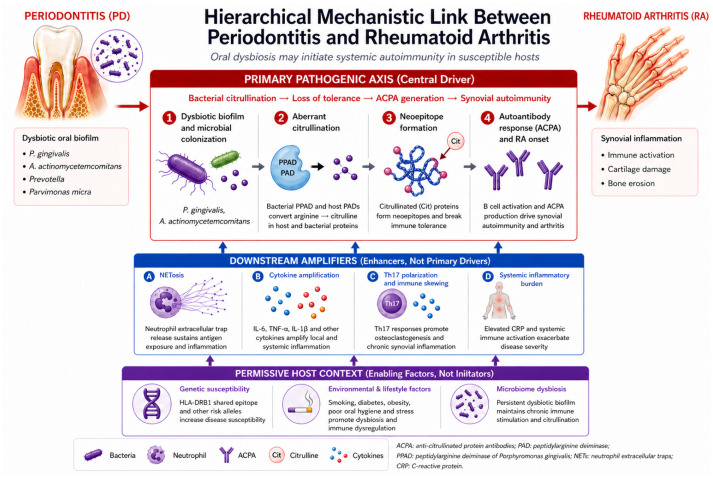
Hierarchical mechanistic framework linking periodontitis (PD) and rheumatoid arthritis (RA). The model proposes that the most plausible upstream biological connection between PD and RA is a central pathogenic axis driven by bacterially-mediated protein citrullination. In dysbiotic periodontal biofilms enriched with key pathobionts, particularly *Porphyromonas gingivalis* and *Aggregatibacter actinomycetemcomitans*, microbial virulence factors promote aberrant citrullination either directly through bacterial peptidylarginine deiminase activity or indirectly through activation of host peptidylarginine deiminases. This process generates citrullinated neoepitopes that break immune tolerance, stimulate anti-citrullinated protein antibody (ACPA) production, and contribute to synovial autoimmunity, inflammation, cartilage damage, and bone erosion characteristic of RA. Secondary downstream amplifiers—including neutrophil extracellular trap (NET) formation, cytokine amplification (e.g., IL-1β, IL-6, TNF-α), Th17 polarization, and systemic inflammatory burden—may further intensify local and systemic immune activation but are not considered primary initiators. At the base of the hierarchy, permissive host-context factors such as genetic susceptibility (e.g., HLA-DRB1 shared epitope), environmental and lifestyle exposures (smoking, diabetes, obesity, poor oral hygiene), and persistent oral microbiome dysbiosis increase susceptibility and modulate disease severity. Collectively, this framework supports a hierarchical model in which periodontal dysbiosis initiates autoimmune priming, while host and inflammatory modifiers govern progression toward clinically manifest RA. Abbreviations: ACPA, anti-citrullinated protein antibodies; NETs, neutrophil extracellular traps; PPAD, *P. gingivalis* peptidylarginine deiminase; PAD, peptidylarginine deiminase; Cit, citrulline; RA, rheumatoid arthritis; PD, periodontitis.

**Figure 4:**
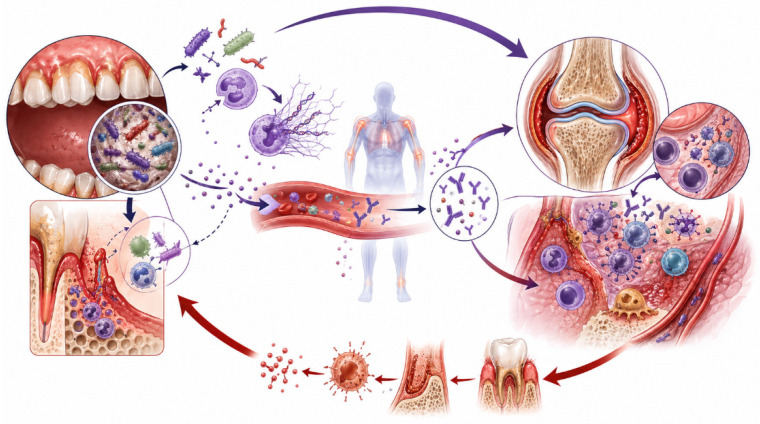
Bidirectional pathophysiological model linking PD and RA. The illustration depicts a circular bidirectional relationship between periodontal inflammation and RA-mediated through microbial, immunological, and systemic inflammatory pathways. On the left, dysbiotic oral biofilm and inflamed periodontal tissues represent PD, characterized by pathogenic bacterial accumulation, gingival inflammation, immune-cell infiltration, and progressive destruction of periodontal connective tissue and alveolar bone. Periodontal pathogens and locally generated inflammatory mediators enter the circulation through transient bacteremia, illustrated centrally by dissemination of microbial products, immune complexes, and soluble mediators through the bloodstream. These circulating factors promote systemic immune activation, loss of tolerance to citrullinated antigens, and autoantibody responses. On the right, these processes contribute to synovial inflammation characteristic of RA, with leukocyte infiltration, pannus formation, cartilage degradation, and bone erosion shown within the affected joint. The lower red feedback loop illustrates the reverse pathway whereby chronic systemic inflammation associated with RA, including cytokine excess and immune dysregulation, may exacerbate periodontal breakdown by enhancing osteoclastogenesis, connective tissue destruction, and susceptibility to further dysbiosis. Together, the figure summarizes a self-reinforcing bidirectional cycle in which PD may contribute to RA initiation or progression, while RA-associated systemic inflammation may worsen periodontal disease severity.

PD and RA are chronic inflammatory and tissue-destructive disorders linked by overlapping genetic susceptibility, microbial dysbiosis, and dysregulated host immunity. However, current evidence does not support equivalent pathogenic relevance across these pathways. Rather, the most compelling and consistently supported mechanistic link converges on a central axis of bacterially driven protein citrullination, subsequent loss of immune tolerance, and generation of ACPA. This model is uniquely supported by converging microbiological, immunological, genetic, and clinical evidence, whereas other proposed mechanisms are more plausibly interpreted as downstream amplifiers or modifiers of this primary autoimmune process (Figure 3 and Figure 4).

Within this central framework, key periodontal pathogens such as *Porphyromonas gingivalis* and *Aggregatibacter actinomycetemcomitans* play a central mechanistic role through their ability to induce protein citrullination. *Porphyromonas gingivalis* expresses a bacterial peptidyl arginine deiminase enzyme that catalyzes the conversion of arginine to citrulline in host and bacterial proteins, thereby altering protein structure, charge, and immunogenicity [36]. This process is further enhanced by gingipains, which generate arginine-containing substrates for citrullination, amplifying the formation of neoepitopes. *Porphyromonas gingivalis* is particularly relevant because it expresses a specific enzyme responsible for citrullination on its outer membrane, the Porphyromonas peptidyl arginine deiminase (PPAD). This bacterial modification generates neoepitopes that stimulate ACPA production in genetically susceptible individuals and enhance autoimmune responses [11]. In contrast, *Aggregatibacter actinomycetemcomitans* induces hypercitrullination indirectly through leukotoxin A-mediated pore formation in neutrophils, leading to calcium influx and activation of host peptidyl arginine deiminase enzymes, particularly PAD4. Both pathways result in the generation of citrullinated autoantigens that promote the production of ACPA, a hallmark of RA [36].

Beyond direct citrullination, these microbial stimuli may further amplify autoimmunity through dysregulated neutrophil responses, particularly neutrophil extracellular trap (NET) formation. NETs are enriched with citrullinated proteins, histones, and antimicrobial enzymes. Although these structures contribute to bacterial clearance, excessive or dysregulated formation leads to collateral tissue damage and amplification of inflammation within periodontal tissues [36]. *Porphyromonas gingivalis* further modulates this response by citrullinating key immune mediators such as complement C5a, chemokines, and cationic antimicrobial peptides, thereby impairing chemotaxis, neutrophil activation, and bacterial clearance. In addition, delivery of peptidyl arginine deiminase via outer membrane vesicles enables intracellular targeting of host components, including histone H3, promoting cytotoxicity and sustained inflammatory signaling [36].

At the broader ecological level, these host–pathogen interactions occur within a dysbiotic oral microbiome that may sustain chronic inflammation and favor persistence of pathogenic species. Microbial dysbiosis within the oral cavity has emerged as a permissive microbial environment associated with the coexistence of PD and RA. Alterations in the oral microbiome, characterized by increased abundance of pathogenic species such as Prevotella, *Aggregatibacter actinomycetemcomitans*, Parvimonas micra, and *Porphyromonas gingivalis*, have been associated with more severe periodontal conditions and RA activity [11].

These processes allow systemic dissemination of citrullinated antigens, reinforcing the pre-existing ACPA-driven autoimmune response between local periodontal dysbiosis and systemic immune dysregulation [36].

*Porphyromonas gingivalis* also contributes to disease progression through additional immunomodulatory pathways. It promotes T helper 17 cell differentiation, activates complement C5a through Rgp activity, induces adhesion to immune cells through FimA, stimulates lipopolysaccharide-mediated cytokine production, and facilitates bacterial translocation to joint tissues. Oral infection may also induce intestinal dysbiosis and a shift toward a T helper 17 dominated immune response [50]. Clinical observations have shown a significant association between the abundance of *Porphyromonas gingivalis* in the tongue biofilm and RA disease activity measured by the Disease Activity Score on 28 joints (DAS28) [7].

*Aggregatibacter actinomycetemcomitans* can induce hypercitrullination in neutrophils through leukotoxin A activity, which alters neutrophil morphology and mimics extracellular trap formation. This process results in the release of hypercitrullinated autoantigens that can trigger autoimmune responses in RA [7]. Experimental studies further show that systemic infection with *Aggregatibacter actinomycetemcomitans* can aggravate arthritis through caspase-11-mediated inflammasome activation in macrophages, leading to interleukin-1β secretion and inflammatory cell infiltration in joint tissues [51]. Dissemination of periodontal pathogens and their antigens may occur through transient bacteremia during routine oral activities. Bacterial DNA from periodontal species, including *Porphyromonas gingivalis*, *Treponema denticola*, Prevotella intermedia, *Tannerella forsythia*, and Fusobacterium nucleatum, has been detected in the synovial fluid of patients with RA, together with elevated antibody titers directed against these microorganisms. These findings support the hypothesis that microbial components originating from periodontal tissues may reach distant anatomical sites and contribute to inflammatory processes within the joints [11].

However, microbial exposure alone is unlikely to fully explain disease transition, as host genetic susceptibility appears to modulate immune responses to these stimuli. Genetic factors also support the biological connection between periodontal disease and RA. The highly polymorphic HLA-DRB1 locus containing the shared epitope represents the strongest genetic factor involved in RA development and has also been associated with periodontal disease susceptibility [7]. Shared epitope-coding DRB1 alleles have been associated with bone erosions in RA as well as alveolar bone destruction during periodontal disease progression [11]. The shared epitope functions as a signal transduction ligand that facilitates Th17 differentiation and osteoclast activation, thereby increasing disease severity [11]. Experimental studies have demonstrated that transgenic mice expressing shared epitope-positive DRB1 alleles spontaneously develop periodontal disease accompanied by IL-17 overexpression and disruption of periostin, along with reduced mandibular bone volume and increased alveolar bone resorption [52]. In addition, the generation of ACPA in response to *Porphyromonas gingivalis* infection requires the expression of PAD enzymes that citrullinate host-derived proteins, suggesting a possible causative link between periodontal infection and RA [11].

Once initiated, these immune responses converge with inflammatory pathways shared by both PD and RA, including elevated IL-1, IL-6, and TNF signaling. Both diseases are characterized by similar inflammatory pathways. Elevated levels of pro-inflammatory cytokines, including IL-1, IL-6, and TNF, have been demonstrated in both RA synovial tissues and periodontal gingival tissues. Increased expression of these mediators can activate STAT3 signaling pathways that play a key role in the pathophysiology of both conditions [7]. Immune cell populations, including neutrophils, monocytes, and lymphocytes, expand in both diseases and produce pro-inflammatory mediators and tissue-degrading enzymes such as matrix metalloproteinases and cathepsins, which contribute to connective tissue destruction and bone resorption. Autoantibody responses also contribute to the association between periodontal disease and RA. Periodontitis-induced NETs can generate carbamylated proteins and anti-carbamylated protein antibodies, which may react with joint tissues and contribute to inflammatory processes leading to RA. At the molecular level, circulating oral bacteria containing citrullinated epitopes can stimulate ACPA-producing B cells and promote antibody affinity maturation and epitope spreading toward citrullinated human proteins, thereby amplifying autoimmune responses and contributing to disease exacerbation [53].

One proposed explanation is the “two-hit” model. In this model, the first hit involves the increased presence of anaerobic microorganisms and their antigens within the periodontal microenvironment. This microbial challenge initiates destructive events characteristic of PD, including increased production of bone-resorptive cytokines such as IL-6, IL-1, and TNF-α, as well as tissue-destructive proteinases including matrix metalloproteinases [54,55]. The second hit involves systemic inflammatory diseases such as RA, which increase circulating biomarkers of inflammation, including CRP, IL-6, IL-1β, PGE2, matrix metalloproteinases, and TNF-α. Elevated systemic inflammatory mediators can further stimulate immune cells within the periodontium, enhancing the production of matrix metalloproteinases and receptor activator of nuclear factor kappa B ligand, thereby aggravating destruction of periodontal connective tissues and bone in a process similar to cytokine-driven osteoclast activation observed in RA pathogenesis [11,54,55].

Collectively, these findings support a hierarchical model in which bacterially induced citrullination constitutes the principal upstream trigger, whereas NETosis, dysbiosis, host genetic susceptibility, and inflammatory amplification pathways modulate disease expression and progression.

### 3.4. Reciprocal Therapeutic Effects

The growing body of evidence supporting the biological and inflammatory connections between RA and PD has led to increasing interest in understanding whether treatment of one condition can influence the course of the other. Both diseases share common pathogenic mechanisms, including dysregulated immune responses, overproduction of pro-inflammatory cytokines, and tissue destruction-mediated by matrix metalloproteinases and osteoclast activation. Consequently, therapeutic interventions targeting systemic or local inflammation may exert reciprocal effects on both conditions.

#### 3.4.1. Effects of Periodontal Treatment on Rheumatoid Arthritis Outcomes

Non-surgical periodontal therapy aims to disrupt the subgingival biofilm in order to control inflammation associated with periodontal disease. According to the European Federation of Periodontology, which established an S3-level guideline based on a stepwise treatment approach, management begins with behavioral modification. This initial step focuses on motivating patients to effectively remove dental biofilm and control modifiable risk factors. The second step involves subgingival instrumentation, including subgingival scaling and root surface debridement, with the objective of eliminating calculus and pathogenic biofilm. Adjunctive therapies may be considered when indicated. The third step includes reassessment and, if necessary, repeated subgingival instrumentation or progression to surgical periodontal procedures when non-surgical therapy alone is insufficient [56].

Several clinical investigations have evaluated whether periodontal therapy can influence RA disease activity and systemic inflammatory markers (Table 3).

A randomized clinical study conducted by Ortiz et al. [57] evaluated forty participants diagnosed with moderate to severe RA and severe PD. All participants were receiving DMARDs, and twenty were also treated with anti– TNF-α agents prior to randomization. The participants were divided into two groups, with twenty patients receiving non-surgical periodontal therapy consisting of scaling and root planing combined with oral hygiene instructions, while the remaining twenty patients received no periodontal treatment. Periodontal parameters, including probing depth, CAL, BOP, GI, and plaque index (PI), were assessed together with RA DAS28 and erythrocyte sedimentation rate (ESR) at baseline and after six weeks. Patients receiving periodontal therapy demonstrated significant reductions in DAS28, ESR (*p* < 0.001), and serum TNF-α levels (*p* < 0.05). In contrast, patients who did not receive periodontal therapy showed no significant changes in these parameters. The study population included patients with at least 20 teeth and excluded smokers, diabetics, and individuals with severe xerostomia. These findings indicate that non-surgical periodontal therapy may improve both periodontal health and RA disease activity, independent of ongoing pharmacological treatment.

Erciyas et al. [58] evaluated sixty patients with RA and chronic PD, divided into low disease activity (DAS28 < 3.2) and moderate to high disease activity (DAS28 ≥ 3.2), all receiving non-surgical periodontal therapy and assessed at baseline and three months. Significant reductions were observed in DAS28, ESR, CRP, serum TNF-α, and all periodontal parameters after treatment, with greater improvement in patients with higher baseline disease activity. These findings indicate that periodontal therapy may reduce systemic inflammation and RA activity, likely through cytokine-mediated pathways involving TNF-α. Confounding factors including DMARDs, corticosteroids, NSAIDs, and anti-TNF-α therapy were present, while oral hygiene, disease duration, and socioeconomic status were not fully controlled. The main limitation is the absence of a non-treated control group and short follow-up period, limiting causal inference and long-term interpretation. Despite this, the study supports a clinically relevant association between periodontal inflammation and systemic autoimmune activity and suggests periodontal therapy as an adjunctive approach in RA management.

Additional evidence supporting the beneficial effects of periodontal treatment was provided by El Wakeel et al. [59], who evaluated eighty participants allocated into four balanced groups including patients with RA and concomitant stage III or IV PD with moderate disease activity defined by DAS28, patients with RA without PD, individuals with PD alone, and healthy controls. All PD cases underwent scaling and root planing and prolactin levels were measured in GCF, serum, and synovial fluid. At baseline, GCF prolactin was highest in the RA with PD group, whereas the lowest levels were observed in RA without PD and in healthy controls, with a statistically significant difference and a large effect size. Following periodontal therapy, GCF prolactin decreased significantly in both PD groups with large effect sizes, although post treatment levels remained higher than those observed in RA without PD and controls. Serum prolactin did not differ significantly among the disease groups at baseline but was higher than in controls, and after therapy it decreased significantly in both PD groups while remaining elevated compared with controls. Synovial fluid prolactin was higher in RA with PD than in RA alone at baseline without statistical significance, and showed a significant reduction after treatment with a large effect size. Periodontal clinical parameters improved markedly after therapy. Study reduced confounding by excluding smokers, individuals with other systemic diseases, those receiving biological DMARDs, and those with recent antibiotic use or periodontal therapy or abnormal body mass index, although residual confounding may persist due to variability in conventional DMARDs regimens, disease duration, oral hygiene behavior, socioeconomic status, and metabolic factors. Overall, these findings support that periodontal therapy may reduces local and partially systemic inflammatory mediators involved in the interaction between PD and RA.

Further supporting evidence comes from systematic review data showing that periodontal treatment is associated with statistically significant improvements in both periodontal and rheumatologic outcomes, including a measurable reduction in CAL and a concurrent decrease in DAS among patients affected by both conditions, reinforcing the hypothesis that control of periodontal inflammation may contribute to reduced RA severity. Interpretation of these findings should consider important confounders including the strong immunomodulatory effects of DMARDs and biologic therapies, inconsistent adjustment for smoking as a shared risk factor, variability in periodontal disease definitions and measurement protocols, and limited control of metabolic comorbidities such as diabetes, all of which may influence both periodontal and rheumatologic outcomes. Additional limitations include exclusion of multimorbid populations, reducing external validity, as well as small sample sizes and short follow up periods, which limit inference on long term causality. Despite these constraints, the generally consistent direction of effect and low heterogeneity for CAL outcomes support the robustness of the association, reinforcing the concept that periodontal inflammation may act as a modifiable contributor to systemic inflammatory burden in RA through cytokine-mediated mechanisms and bacterial dissemination, and that periodontal therapy may contribute to a reduction in disease severity [60].

However, not all investigations demonstrate clear improvements. Huang et al. [61] analyzed randomized controlled trials comparing scaling and root planing in patients with RA and PD versus non-RA individuals with PD. Overall, no statistically significant differences were observed in probing depth reduction, plaque index, GI, CAL, or BOP between groups, indicating that RA does not impair the overall clinical response to non-surgical periodontal therapy. However, subgroup analyses showed transient improvements in BOP at early follow up and modest benefits in plaque control and CAL at later follow up, suggesting time dependent inflammatory variability rather than sustained differential treatment effects. The evidence is limited by the small number of trials, modest sample sizes, heterogeneous follow up periods, and incomplete reporting of RA disease duration, which may act as an important confounder, together with signals of heterogeneity and potential publication bias that reduce certainty of the findings. Overall, the study supports comparable periodontal treatment outcomes in RA and non-RA patients, while emphasizing the need for larger, well controlled longitudinal trials incorporating standardized measures of RA activity and disease duration.

Several clinical investigations have evaluated the impact of advanced periodontal therapies, including laser-based approaches, on RA-related clinical and inflammatory outcomes.

Some clinical data suggest that laser acupuncture may reduce DAS28 and systemic inflammation, including decreased CRP, IL-6, and ESR, along with improved antioxidant status such as increased SOD, glutathione, and catalase activity [62]. Low-level laser therapy (LLLT) has also been reported to improve functional parameters such as grip strength and morning stiffness, although effects on pain and overall clinical outcomes remain unclear [63]. However, systematic evidence from randomized trials indicates low-certainty findings, with no significant benefit of infrared or red laser therapy over sham for pain, inflammation, or disease activity in RA [64]. Preclinical models suggest that low-level laser irradiation may reduce synovial inflammation by decreasing TNF-α and fibroblast-like synoviocyte activity while increasing synovial apoptosis [65]. In addition, laser-based analytical technologies have shown potential for sensitive metabolic profiling [66]. Overall, current evidence suggests possible biological and symptomatic effects; however, to our knowledge, there is no robust clinical evidence demonstrating consistent improvement in RA clinical or inflammatory parameters following advanced periodontal laser therapies.

Systemic tetracycline therapy (minocycline) has shown consistent benefits in RA, including improvement in joint swelling and tenderness, and reductions in inflammatory markers such as ESR, platelet count, IgM, and RF compared with placebo [67]. In early seropositive RA, minocycline also resulted in higher ACR50 response rates and reduced corticosteroid requirements compared with hydroxychloroquine [68], and observational data suggest altered disease activity profiles in treated patients [69]. These effects may be partly related to antimicrobial activity against periodontal pathogens and modulation of citrullination-driven autoimmunity, supported by evidence that eradication of *Aggregatibacter actinomycetemcomitans* infection can resolve arthritis and normalize ACPA levels in a reported case [70].

Overall, current evidence suggests that systemic antimicrobial therapy may influence RA disease activity, whereas, to our knowledge, the impact of advanced local periodontal therapies on RA-related outcomes remains unproven and requires further investigation. Importantly, no clinical studies have directly evaluated the effects of locally delivered periodontal antimicrobials on RA clinical or inflammatory outcomes.

Although some studies discussed above have shown that periodontal treatment may improve RA, it is important to note that neither the RA population nor periodontal treatment represents a homogeneous or uniform condition. People with RA, as discussed in Section 3.1.3, are characterized by different disease stages and durations. It is also important to emphasize that patient stratification, particularly according to ACPA status, may plays a central role in defining pathogenic mechanisms and therapeutic responses.

There is evidence suggesting that the RA phenotype, particularly autoantibody status, is closely linked to periodontal disease severity and inflammatory burden. Patients with RA who are ACPA or anti-CCP positive consistently present with more severe PD and a more pronounced inflammatory and microbial profile compared with seronegative individuals [71]. Similarly, anti-CCP positivity has been independently associated with PD severity in RA populations, even after adjustment for major confounders [72]. In addition, both RF and anti-CCP positivity have been significantly associated with moderate to severe PD, whereas no consistent relationship has been observed with other measures of RA disease activity, suggesting that serological phenotype may be more relevant than clinical disease activity in periodontal involvement [27]. Furthermore, periodontal severity parameters, including CAL, plaque index, and number of deep periodontal pockets, have been shown to correlate linearly with anti-CCP antibody levels, supporting a dose-dependent relationship between autoimmunity and periodontal destruction [73].

From a mechanistic perspective, these observations are biologically plausible. *Porphyromonas gingivalis*, a key periodontal pathogen, directly expresses peptidylarginine deiminase, which enables citrullination of bacterial and host proteins, generating neoantigens that may trigger ACPA production and perpetuate systemic autoimmunity [74,75]. Furthermore, periodontal pathogens have been shown to modulate host immune responses, promote neutrophil dysfunction, and sustain chronic inflammation, thereby providing a potential link between periodontal infection and autoimmune activation in RA [36]. These mechanisms suggest that citrullination-driven pathways and microbial burden may be more pronounced in ACPA-positive individuals, potentially leading to differences in host immune response and disease expression between seropositive and seronegative patients. The ability of this microorganism to express peptidyl arginine deiminase enables direct citrullination of host proteins, leading to the generation of neoepitopes and amplification of autoimmune responses in genetically predisposed individuals. This mechanism is particularly relevant in ACPA positive patients, in whom citrullinated antigens play a pivotal role in disease pathogenesis. In contrast, ACPA negative RA may involve alternative inflammatory pathways that are less dependent on citrullination, suggesting that the contribution of periodontal dysbiosis may differ substantially between these subgroups.

However, to our knowledge, no clinical studies have directly evaluated whether periodontal treatment outcomes differ according to AR serological phenotype, particularly ACPA-positive versus ACPA-negative disease, representing an important gap in the current literature. Consequently, it remains unknown whether seropositive and seronegative patients exhibit differential clinical responses to periodontal interventions such as scaling and root planing. Similarly, the extent to which DMADs may differentially influence periodontal outcomes according to the underlying immunological phenotype has not been established. Collectively, these unresolved questions underscore the need for well-designed, phenotype-stratified prospective studies to clarify therapeutic interactions, identify biologically distinct treatment responses, and support more personalized management strategies for patients with coexisting RA and periodontal disease.

#### 3.4.2. Effects of Rheumatoid Arthritis Therapy on Periodontal Condition

RA management aims to achieve rapid disease control, relieve symptoms, prevent joint damage, and improve quality of life. The principle of “treat-to-target,” as recommended by the American College of Rheumatology (ACR), emphasizes early, aggressive intervention to achieve remission or low disease activity, as established erosions are irreversible. Optimal care integrates accurate diagnosis, prevention strategies, non-pharmacological measures, and pharmacological therapies [18].

Non-pharmacological measures—including lifestyle modifications, physical exercise, occupational therapy, rest, and complementary approaches such as massage, thermal therapy, acupuncture, and progressive muscle relaxation—contribute to improved mobility, reduced pain, and enhanced quality of life. Omega-3 polyunsaturated fatty acids (DHA and EPA) may reduce inflammation, pain, and mood disturbances, although further studies are required. Joint surgery is reserved for advanced disease to restore function and relieve pain [18].

Pharmacological management includes symptomatic therapy with nonsteroidal anti-inflammatory drugs (NSAIDs) and glucocorticoids (GCs), as well as disease-modifying antirheumatic drugs (DMARDs). DMARDs, which suppress autoimmune activity and slow joint degeneration, are classified as conventional synthetic (csDMARDs), biologic (bDMARDs), and targeted synthetic (tsDMARDs, including Janus kinase inhibitors, JAKi). Methotrexate is the recommended first-line csDMARD due to its efficacy, safety, and cost-effectiveness, with leflunomide, hydroxychloroquine, and sulfasalazine as alternatives. Biologic agents, including TNF-α inhibitors (etanercept, infliximab, adalimumab, golimumab, certolizumab), B-cell-targeted therapies (rituximab), T-cell modulators (abatacept), and cytokine inhibitors (IL-1, IL-6, IL-12/23, IL-17), provide targeted immunomodulation for patients inadequately responsive to csDMARDs. Clinical trials and post-marketing studies demonstrate their efficacy in reducing disease activity and radiographic progression, although risks include serious infections and, for some TNF-α inhibitors, tuberculosis [18].

JAK inhibitors, a newer class of orally administered tsDMARDs, offer selective modulation of cytokine signaling and represent a cost-effective alternative to biologics. Approved agents such as tofacitinib, baricitinib, and upadacitinib have demonstrated efficacy in moderate-to-severe RA, with safety profiles generally consistent with their immunosuppressive mechanism, although monitoring for infections, thromboembolic events, and lipid alterations is required [18].

Pharmacological treatment of RA has also been investigated for its potential impact on periodontal health. Antirheumatic medications target key inflammatory pathways involved in both RA and periodontal tissue destruction, suggesting that systemic therapy may influence periodontal inflammation. Several clinical investigations have evaluated whether systemic antirheumatic therapy may influence periodontal inflammation (Table 4).

Hatipoğlu et al. [76] conducted a case–control study including seventy participants, consisting of forty patients with RA and thirty healthy controls. Among the RA patients, twenty were undergoing B-cell depletion therapy, while twenty were receiving DMADs without B-cell depletion. Rheumatologic parameters, including DAS28, RF, and anti-cyclic citrullinated peptide levels, were also analyzed. Periodontal parameters, including probing depth, CAL, BOP, GI, and plaque index, were recorded. In addition, levels of interleukin-1β and matrix metalloproteinase-8 in GCF were measured using enzyme-linked immunosorbent assay. Although periodontal clinical parameters were similar across groups, GCF IL-1β levels were significantly lower in the B-cell depletion group compared with the DMARD and control groups (*p* < 0.001), while MMP-8 levels were significantly lower in the DMARD group (*p* < 0.001). These results suggest that RA treatments may modify inflammatory biomarkers within the periodontal environment, potentially contributing to reduced periodontal tissue destruction [63]. This study provides mechanistic evidence linking systemic immunomodulation in RA to local periodontal inflammatory mediators. The pronounced reduction in IL-1β in the B-cell depletion group supports a potential role of B cells in periodontal cytokine production, although causality cannot be inferred due to the cross-sectional design. A major limitation is the absence of untreated RA patients, making it difficult to isolate disease versus drug effects. Additionally, periodontal clinical parameters were similar across groups, suggesting that biochemical changes may not yet translate into measurable clinical improvement. This discrepancy may reflect the inability of systemic therapy alone to adequately control local periodontal biofilm-driven inflammation, emphasizing the need for adjunctive periodontal treatment. Small sample size and single-time-point measurement further limit generalizability. Nevertheless, the study strengthens the concept of shared immunoinflammatory pathways between RA therapy and periodontal host response modulation.

In the cross-sectional comparison, oral hygiene behavior and caries experience were comparable between patients exposed to B-cell–depleting therapy and those not exposed, suggesting that baseline differences in oral hygiene were unlikely to explain periodontal differences. Despite similar general oral health behaviors, the group receiving rituximab exhibited a more favorable periodontal profile, characterized by reduced clinical signs of gingival inflammation and lower measures of periodontal tissue breakdown. Importantly, these differences were observed in the context of a lower plaque burden in the non-treated group, indicating that improved periodontal status in the treated group was not attributable to better oral hygiene control. Collectively, these findings support a potential protective effect of B-cell depletion on periodontal tissues independent of conventional local risk factors. The longitudinal analysis further reinforced these observations. Following initiation of B-cell–targeted therapy, gingival inflammatory indices showed stability or modest improvement, while parameters reflecting connective tissue attachment and periodontal pocketing demonstrated consistent reduction. Importantly, these structural improvements occurred without meaningful changes in oral hygiene behavior, suggesting that the observed periodontal benefits were not driven by behavioral modification but rather by systemic immunomodulation. Over extended follow-up, individuals repeatedly exposed to therapy maintained or further improved their periodontal condition, with a tendency toward stabilization of previously progressive lesions, including in cases of more advanced periodontal involvement. From a mechanistic perspective, these findings are consistent with the hypothesis that B lymphocytes play a central role in the immunopathogenesis of periodontal tissue destruction. The observed clinical stabilization supports the concept that B-cell–mediated immune activity contributes not only to inflammatory amplification but also to downstream activation of osteoclastogenic pathways implicated in alveolar bone loss. The data align with experimental and translational evidence linking B-cell activity with periodontal breakdown and suggest that targeted depletion may shift the host response toward a less destructive inflammatory phenotype. Importantly, the study also highlights a dissociation between inflammatory and structural periodontal outcomes, suggesting that modulation of specific immune pathways may preferentially influence tissue destruction while exerting a more limited effect on gingival inflammation in the short term. This pattern is consistent with previous observations in related immunomodulatory approaches and underscores the complexity of inflammatory resolution in chronic periodontal disease. Overall, these results provide clinically evidence supporting a possible potential therapeutic role of B-cell–targeted immunotherapy in modifying periodontal disease progression, particularly in systemic inflammatory conditions such as RA. However, given the small sample size and observational nature of the analysis, these findings should be interpreted as hypothesis-generating and warrant validation in larger controlled studies designed specifically to evaluate periodontal endpoints [77].

A systematic review and meta-analysis conducted by Zhang et al. [78] provides moderate-quality evidence that anti-rheumatic therapy in patients with RA and PD is associated with statistically significant improvements in periodontal parameters, particularly probing depth, CAL, and gingival inflammation indices. The magnitude of effect is modest but consistent across pooled outcomes, suggesting a biologically plausible interaction between systemic immunomodulation and periodontal tissue response. However, the evidence is constrained by substantial clinical and methodological heterogeneity, including variability in anti-rheumatic drug classes, differences in baseline periodontal severity, inconsistent diagnostic criteria for PD, and limited sample size within the meta-analytic component. These factors reduce the precision of effect estimation and limit direct clinical generalizability. Importantly, the observed improvements are not uniform across all inflammatory markers, with gingival inflammation showing variability depending on study design and treatment duration, highlighting the complexity of cytokine-driven periodontal responses in systemic autoimmune disease. Additionally, confounding factors such as smoking status, disease duration, and concurrent periodontal therapy were not consistently controlled across included studies. Overall, while anti-rheumatic therapy appears to confer a supportive benefit on periodontal health in RA patients, the effect should be interpreted as adjunctive rather than therapeutic for PD itself. Robust longitudinal randomized studies with standardized periodontal definitions and stratified biologic therapies are required to clarify causality and magnitude of clinical benefit.

This longitudinal comparative study suggests that targeting IL-6 signaling via tocilizumab exerts a measurable beneficial effect on periodontal inflammation in patients with RA and PD. While both IL-6 and TNF inhibition reduced classical inflammatory periodontal parameters (GI, BOP, Probing depth), only IL-6 blockade was associated with a significant gain in CAL, indicating a potential advantage in stabilizing periodontal tissue destruction rather than only suppressing inflammation. Importantly, plaque levels remained unchanged across groups, strengthening the interpretation that observed improvements are driven by systemic immunomodulation rather than improved oral hygiene behavior. However, the study is limited by its non-randomized design, modest sample size, and absence of reported standardized effect sizes or confidence intervals, which restricts quantitative comparability for meta-analysis. Additionally, potential confounding from concurrent RA medications (DMARDs, corticosteroids) cannot be fully excluded, although treatment regimens were stable during follow-up. Overall, the findings support a biologically plausible IL-6–driven inflammatory axis linking RA and PD and suggest that IL-6 receptor inhibition may offer slightly enhanced periodontal benefit compared with TNF blockade, particularly in terms of connective tissue preservation [79].

Nevertheless, not all studies report improvements in periodontal status following anti-rheumatic therapy. A longitudinal observational study by de Smit evaluated the effects of methotrexate and anti–tumor necrosis factor therapy on the periodontal condition of patients with RA. The findings indicate that, although these therapies significantly reduce systemic inflammatory burden, they do not translate into measurable improvements in periodontal inflammation. The absence of significant changes in PISA, despite reductions in CRP and ESR, suggests a dissociation between systemic and local inflammatory responses. Importantly, the lack of periodontal treatment during the study is a major confounder, as biofilm-driven inflammation remains unaddressed. The small sample size, non-randomized design, and reliance on median-based non-parametric analysis further limit statistical power and generalizability. Additionally, the absence of reported effect sizes, confidence intervals, and detailed periodontal parameter statistics (BOP, Probing depth, CAL) reduces interpretability and prevents meta-analytic inclusion without assumptions. Overall, the study supports the concept that systemic immunosuppression alone is insufficient to control periodontal disease, emphasizing the necessity of local periodontal therapy in RA patients [80].

Additional research indicates that specific antirheumatic medications may influence the periodontal microbiological environment.

The cross-sectional study by Romero-Sánchez et al. [81] demonstrates that different anti-rheumatic treatment regimens exert heterogeneous effects on periodontal status and subgingival microbiota in patients with RA. Anti-TNF therapy combined with methotrexate was associated with reduced CAL; however, it also altered the microbial profile, notably increasing the prevalence of *Treponema denticola*. Corticosteroid therapy was similarly associated with a protective effect on tooth retention. Furthermore, elevated anti–cyclic citrullinated peptide (anti-CCP) antibody titers were associated with the presence of red complex periodontal pathogens, whereas increased C-reactive protein (CRP) and erythrocyte sedimentation rate (ESR) were correlated with greater probing depth and BOP. These associations between systemic inflammatory markers and periodontal parameters further support the bidirectional relationship between RA and PD. However, the cross-sectional design precludes causal inference, and the absence of effect size estimates and confidence intervals limits quantitative interpretation. Overall, the findings suggest that systemic RA therapies alone are insufficient to consistently improve periodontal health and may differentially influence both microbial composition and clinical periodontal outcomes.

Despite generally favorable trends, the impact of antirheumatic therapy on periodontal outcomes remains heterogeneous across studies. Several factors may account for these discrepancies. First, differences in patient characteristics, including disease duration, baseline periodontal severity, smoking status, oral hygiene practices, age, and sex, may significantly influence periodontal responses. Second, variability in RA phenotype, particularly ACPA-positive versus ACPA-negative status, may modulate systemic inflammatory burden and periodontal susceptibility, as discussed in Section 3.4.1. Third, therapeutic heterogeneity is likely to play a central role. Biologic agents, particularly B-cell depletion therapies such as rituximab and IL-6 inhibitors such as tocilizumab, appear to exert more pronounced effects on periodontal inflammation compared with conventional synthetic DMARDs, likely due to their targeted modulation of key cytokines implicated in both RA and periodontal disease. In contrast, therapies such as methotrexate may effectively reduce systemic inflammation without producing equivalent effects on local periodontal tissue responses.

Additionally, heterogeneity in study design, including small sample sizes, short follow-up periods, exclusion criteria related to tooth number, and variability in periodontal assessment methods, further contributes to inconsistent findings. These observations suggest that systemic inflammatory control alone may not be sufficient to consistently improve periodontal outcomes without concomitant local periodontal therapy. It is also important to emphasize that overlapping autoimmune conditions, particularly secondary Sjögren’s syndrome, may further modify periodontal status through xerostomia and consequent alterations in the oral microbiome, thereby acting as an additional confounding factor.

Overall, current evidence suggests that periodontal therapy may be associated with modest improvements in systemic inflammatory markers and RA disease activity. Conversely, RA treatment may also influence periodontal health. Antirheumatic therapies target inflammatory pathways implicated in both RA pathogenesis and periodontal tissue destruction, raising the possibility that systemic treatment may attenuate periodontal inflammation and potentially slow periodontal disease progression. Nevertheless, the reported benefits have generally been modest and inconsistent. Interpretation of the available evidence is further limited by small sample sizes, short follow-up durations, the potential influence of residual confounding, and substantial heterogeneity in study populations and outcome measures.

Taken together, these observations support a possible bidirectional relationship between oral and systemic inflammation and highlight the importance of coordinated, interdisciplinary management strategies integrating rheumatologic and periodontal care.

### 3.5. Collaboration Between Rheumatologists and Dental Professionals

Effective management of patients with RA requires close collaboration between dental professionals and rheumatologists. RA is a chronic systemic inflammatory disease that may present with oral and orofacial manifestations, including periodontal inflammation and temporomandibular joint disorders. Given the bidirectional relationship between systemic inflammation and oral health, a multidisciplinary approach is essential to achieving optimal patient outcomes.

Dentists and dental hygienists play an important role not only in the management of oral conditions but also in the early recognition of signs and symptoms suggestive of undiagnosed RA. A comprehensive medical history and careful clinical assessment during routine dental visits may reveal persistent joint pain, prolonged morning stiffness, swelling of small joints, or reduced manual dexterity that can impair oral hygiene practices. Recognition of these features should prompt timely referral to a rheumatologist, facilitating early diagnosis and initiation of therapy, both of which are critical for improving long-term outcomes. Moreover, effective rheumatologic control of RA disease activity may help reduce the risk of periodontal progression by attenuating systemic inflammation.

Conversely, regular periodontal care and professional maintenance may reduce systemic inflammatory burden and potentially contribute to improved RA disease control. Dental professionals are therefore central to monitoring oral health, implementing preventive strategies, and managing periodontal and temporomandibular complications, while rheumatologists oversee systemic evaluation and pharmacological treatment.

Continuous communication and coordinated care between these disciplines enable a comprehensive understanding of the patient’s condition, support evidence-based therapeutic decision-making, and enhance patient adherence and confidence. Integrated medical and dental management may facilitate early detection, prevent complications, and improve quality of life in individuals living with RA.

## 4. Conclusions

PD and RA are chronic inflammatory disorders that appear to be interconnected through overlapping microbial, immunological, and genetic mechanisms. Current evidence supports a possible bidirectional association in which each condition may influence the onset, severity, or progression of the other. Several studies have reported that periodontal treatment may be associated with improvements in RA activity, while effective rheumatologic therapy may also positively influence periodontal status. These findings suggest that management of one disease may have beneficial effects on the other within a shared inflammatory network.

However, interpretation of these observations requires considerable caution. The available literature is characterized by substantial heterogeneity in study populations, including differences in age, smoking exposure, metabolic comorbidities, socioeconomic background, oral hygiene practices, and treatment history. In addition, both RA and PD are heterogeneous disorders, and outcomes may vary according to disease stage, duration, severity, activity, and immunological phenotype. Variability in diagnostic criteria, periodontal case definitions, therapeutic protocols, and follow-up duration further limits direct comparability across studies.

Multiple confounding factors must also be considered. Immunosuppressive therapies may alter clinical signs of periodontal inflammation, increased healthcare surveillance may introduce detection bias, and overlapping conditions such as secondary Sjögren’s syndrome with xerostomia may independently increase susceptibility to periodontal disease. Moreover, much of the current evidence is derived from observational studies or small interventional cohorts, which limits causal inference and increases susceptibility to residual confounding and selection bias.

Taken together, the relationship between PD and RA is likely multifactorial and embedded within a broader systemic inflammatory milieu rather than representing a simple one-to-one causal interaction. While treatment of one condition may beneficially influence the other, the strength and clinical significance of this effect remain to be fully established. Future research should prioritize large, well-controlled prospective and randomized studies using standardized definitions and phenotype-stratified approaches to clarify causal pathways and guide integrated therapeutic strategies.

## Figures and Tables

**Table 1:** Clinical and epidemiological evidence linking periodontitis to rheumatoid arthritis.

Study	Design	Population	Exposure	Comparator	Outcome	Effect Size	95% CI	*p*-Value	Key Finding
Chou et al. 2015 [19]	Nationwide population-based retrospective cohort	*n* = 894,012 (PD 628,628; DS 96,542; non-PD 168,842), Taiwan	PD	Non-PD; Dental scaling (DS)	Incident RA	Adjusted HR (PD vs. non-PD): 1.89 Adjusted HR: (DS vs. non-PD): 1.43	1.56–2.29 1.09–1.87	<0.001 (both)	PD is associated with significantly increased risk of RA in a dose-dependent manner, independent of age, sex, and diabetes history
Qiao et al. 2020 [20]	Systematic review and meta-analysis	706,611 PD; 349,983 controls	PD	Non-PD	RA risk	OR = 1.69	1.31–2.17	<0.0001	PD significantly increases RA risk (~69% higher odds vs. controls)
Eezammuddeen et al. 2023 [21]	Systematic Review	RA patients	PD	RA without PD	ACPA positivity RF positivity	OR = 1.82 OR = 1.53	1.13–2.93 1.05–2.24	0.01 0.03	RA patients with PD have significantly higher odds of being ACPA-positive. RF positivity: Significant but weaker association than ACPA
Posada-López et al. 2023 [22]	Cross-sectional study	*n* = 75 (21 PD without RA; 33 with RA; 21 reduced periodontium with RA)	Periodontal parameters (CAL, Probing depth, BOP, PIBI)	Non- PD, reduced PD in RA patients	RA presence and biomarkers (ACP, RF, CRP)	NR for PD-RA association	NR for PD-RA association	>0.05 for association with RA diagnosis	No significant association between PD and RA; periodontal parameters were negatively correlated with RA biomarkers.

Abbreviations: RA = rheumatoid arthritis; PD = periodontitis; *n* = number; DS = dental scaling; HR = hazard ratio; OR = odds ratio; CI = confidence interval; CAL = clinical attachment loss; BOP = bleeding on probing; PIBI = Periodontal Inflammatory Burden Index; NR = not reported; RF = rheumatoid factor; ACPA = anti-citrullinated protein antibodies; CRP = C-reactive protein; ACP = anti-citrullinated peptide.

**Table 2:** Evidence supporting the impact of rheumatoid arthritis on periodontal disease development and progression.

Study	Design	Population	Exposure	Comparator	Outcome	Effect Size	95% CI	*p*-Value	Key Finding
Bolstad et al. 2023 [23]	Nationwide registry-based cohort with Cox regression	324,232 total	RA defined by ICD-10 codes and number of visits	Non-RA Controls (fracture/osteoarthritis)	PD based on reimbursement codes	HR = 1.44 (≥10 RA visits vs. no RA, adjusted)	1.35–1.54	<0.001	RA is associated with an increased risk of PD, with a dose–response relationship driven by disease activity, and strongest effects observed in active and newly diagnosed RA patients
Xiao et al. 2021 [24]	Cross-sectional observational	307 RA, 324 non-RA	RA	Healthy individuals without RA	Incidence of PD	51.5% vs. 31.2%	NR	*p* < 0.05	RA patients show significantly higher prevalence of PD than healthy controls. PD severity increases with RA disease duration and age. RA + PD patients exhibit significantly elevated GCF inflammatory cytokines (IL-1β, TNF-α). Systemic inflammation (CRP) and local inflammation (TNF-α) independently predict IL-1β levels in periodontal crevicular fluid
Choi et al. 2016 [25]	Prospective cross sectional	264 RA vs. 88 Non-RA	RA	Non-RA	Prevalence of Moderate–severe PD	63.6% vs. 34.1% OR: 3.38	2.5–4.57	<0.001	prevalence of moderate and severe PD was significantly higher in patients with RA compared with non-RA controls.
González et al 2022 [26]	Cross-sectional observational	110 RA patient with chronic PD	RA disease activity (DAS28 ≥ 4.1) and presence of rheumatic nodules	RA patients with low disease activity vs. high disease activity, and without vs. with rheumatic nodules	Severity of PD Rheumatoid nodules	DAS28 ≥ 4.1 OR = 51.4 OR = 6.4	DAS28 ≥ 4.1 9.4–281.5 1.3–31.6	*p* < 0.0001	RA patients with longer disease duration, higher disease activity and with rheumatic nodules had significantly greater PD severity. Disease activity and rheumatic nodules were strongly associated with severe PD.
Dissick et al. 2010 [27]	Observational cross-sectional pilot study	69 RA 35 osteoarthritis (OA)	RA diagnosis and serological status: RF ACAP	Osteoarthritis group Within RA: RF positive vs. RF negative ACCP positive vs. negative	Presence and severity of PD	Association between PD severity and RA: OR = 2.06	1.11–3.83	*p* = 0.02	PD was more common and severe in patients with RA compared to patients with OA. Patients with RA who were seropositive for were more likely to have moderate to severe PD than patients who were RF negative. Likewise, patients with RA who were positive for the ACPA were more likely to have moderate to severe PD (56%) than patients who were ACCP negative (22%) (*p* = 0.01). There were no associations of PD status with other measures of RA disease activity or severity.
Äyräväinen et al. 2017 [28]	Prospective follow-up study	53 early DMARD-naïve RA, 28 chronic RA with insufficient response to DMARD, 43 controls	Presence of RA (early DMARD-naïve RA and chronic RA with inadequate response to conventional DMARDs)	Non-RA	Presence and Degree of PD, Prevalence of periodontal bacteria rheumatological status by Disease Activity Score, 28-joint count (DAS28)	Early RA vs. controls: OR = 3.6 Chronic RA vs. controls: OR = 5.3	Early RA: 1.1–11.6 Chronic RA: 1.1–25.6	Early RA vs. controls: *p* = 0.036 Chronic RA vs. controls: *p* = 0.044	Patients with RA, both early and chronic forms, exhibit a significantly higher likelihood of moderate PD and worse periodontal status compared with matched controls, and this impairment persists despite treatment with synthetic or biological DMARDs.
Bonilla et al.2025 [29]	Case–control	46 participants (RA *n* = 32; controls *n* = 14)	RA	Non-RA healthy controls	Atherogenic cardiovascular risk profile assessed by lipid parameters Association with PD	NR	NR	PD prevalence higher in RA group 62.5% versus 28.5% BOP higher in RA group *p* < 0.001. Association between periodontal inflammation and LDLC *p* = 0.031 Association between periodontal severity and LDLC *p* = 0.018 Association between periodontal severity and HDLC *p* = 0.003 Associations with CRI 1 and CRI 2 *p* < 0.001.	RA and PD show a synergistic association with increased atherogenic cardiovascular risk with periodontal inflammation and severity linked to adverse lipid profile rather than acting as independent conditions
Juan et al. 2022 [30]	Retrospective cohort study with secondary data analysis	1337 adults with RA	Diagnosis of RA	Non-RA	Incidence of dentist visits for dental disorders including, dental caries, pulpitis, gingivitis, PD and oral ulceration	Adjusted incidence rate ratio(IRR) Gingivitis IRR 1.13 PD IRR 1.13	Gingivitis 1.01 to 1.25 PD 1.04 to 1.22	Gingivitis = 0.027 PD = 0.004	RA is associated with a significantly increased incidence of multiple dental disorders and higher utilization of dental care, with consistent elevation across caries, pulpitis, gingivitis, PD, and oral ulceration
Bonilla et al. 2026 [31]	Observational case–control pilot study	33 RA patients, 22 controls	RA with salivary immune markers (CD11b, CD38, HLA-DR)	Non-RA controls Healthy individuals + degenerative chronic joint	Salivary CD11b/CD38/HLA-DR and periodontal parameters (BOP, PI, PPD, CAL, PIRIM)	Ra vs. control CD11bMFI 45,088 ± 7394 vs. 28,082 ± 22,882 correlated with BOP (r = 0.49), PI (r = 0.35), PIRIM (r = 0.39); ACPA–BOP (r = 0.44); HLA-DR–teeth (r = 0.54)	0.043	CD11b (0.043), CD38 (0.002), key correlations *p* = 0.035–0.047 range; trend for PD (0.069)	RA is associated with higher salivary inflammatory markers, especially CD11b, which correlates with periodontal inflammation, supporting a shared inflammatory pathway with PD.
Pischon et al. 2008 [32]	Cross sectional case–control	57 RA and 52 healthy controls	RA	Non-RA	PD defined as mean clinical attachment loss greater than 4 mm	Adjusted OR = 8.05	2.93–22.09	NR	RA is independently associated with significantly higher odds of PD and this association is only partially explained by oral hygiene measures including plaque index and gingival index
Kim et al. 2019 [33]	Population-based cross-sectional	157 RA 20,140 Non-RA controls	RA	Non-RA	PD	Adjusted odds ratio approximately 0.88	0.57 to 1.36	NR	RA was not associated with PD after controlling for confounders. RA was associated with increased tooth loss only in younger adults under 60 years. No association was observed in older adults.
Susanto et al. 2013 [34]	cross sectional matched case control	75 RA 75 Non-RA	RA	Non-RA	PD prevalence/severity	NS	NR	NS	RA is not associated with increased prevalence or overall severity of PD

Abbreviations: RA = rheumatoid arthritis; PD = periodontitis; HR = hazard ratio; OR = odds ratio; CI = confidence interval; NR = not reported; NS = not significant; GCF = gingival crevicular fluid; DAS = Disease Activity Score; ESR = erythrocyte sedimentation rate; RF = rheumatoid factor; ACPA = anti-citrullinated protein antibodies; ICD code = International Classification of Diseases code; IL = interleukin; CRP = C-reactive protein; TNF = tumor necrosis factor; anti-CCP = anti-cyclic citrullinated peptide antibody; DMARD = disease-modifying antirheumatic drug; LDL-C = low-density lipoprotein cholesterol; HDL-C = high-density lipoprotein cholesterol; CRI = cardiovascular risk index; HLA-DR = human leukocyte antigen–DR; BOP = bleeding on probing; PI = plaque index; PPD = probing pocket depth; CAL = clinical attachment loss; PIRIM = periodontal inflamed risk index measure; CD11bMFI = CD11b mean fluorescence intensity.

**Table 3:** Clinical evidence on the impact of periodontal therapy on rheumatoid arthritis disease activity and inflammatory markers.

Study	Design	Population	Intervention	Comparator	Outcomes	Effect Size	95% CI	*p*-Value	Key Finding
Ortiz et al. 2009 [57]	Randomized controlled clinical	*n* = 40 active moderate to severe RA under treatment + generalized severe chronic PD	Non-surgical periodontal therapy (SRP + OHI)	No periodontal treatment	DAS28 ESR CAL Probing depth	DAS28:Periodontal therapy without anti TNF alpha 5.09 to 3.51; with anti TNF alpha 4.96 to 3.54. ESR: Periodontal therapy without anti TNF alpha 52.5 to 10.5; with anti TNF alpha 51.5 to 22.5. CAL: Periodontal therapy without anti TNF alpha 3.53 to 3.40; with anti TNF alpha 3.82 to 3.52; no significant change without periodontal therapy Periodontal therapy without anti TNF alpha 3.06 to 2.85; with anti TNF alpha 3.25 to 2.82; no significant change without periodontal therapy	NR NR NR NR	0.005 SR 0.640 Less than 0.001 Less than 0.001	Non-surgical periodontal therapy improves signs and symptoms of RA independent of medication use; anti TNF alpha therapy alone does not improve PD status
Erciyas et al. 2013 [58]	Prospective observational cohort	30 moderate to high disease activity (DAS28 3.2) RA and chronic PD 30 low disease activity RA (DAS28 < 3.2) and chronic PD	Non-surgical periodontal therapy (SRP + OHI)	Baseline status within low and high RA activity groups	DAS28, ESR, CRP, serum TNF alpha, CAL, BOP, PI	DAS28: high group 6.25 to 3.94; low group 3 to 2.76 ESR: high group 39.83 to 20.30; low group 13.93 to 11.03 CRP: high group 17.0 to 8.0; low group 3.30 to 3.00 TNF alpha: high group 38.36 to 13.22; low group 30.36 to 11.83 Periodontal parameters PD CAL BOP PI all significantly improved in both groups	NR	Within group all outcomes *p* < 0.05 to *p* < 0.001 Between group differences significant for disease activity and inflammatory reduction *p* < 0.001 for DAS28 ESR CRP	Non-surgical periodontal treatment may prove beneficial in reducing RA severity as measured by ESR, CRP, TNF-a levels in serum and DAS28 in low or moderate to highly active RA patients with chronic PD.
El Wakeel et al. 2023 [59]	Controlled clinical trial (4 groups, pre–post periodontal therapy)	Total 80 (1) 20 RA with moderate activity (DAS28) and PD, (2) 20 RA only, (3) 20 PD only, (4) 20 healthy controls	non-surgical periodontal therapy in patients with PD	RA + PD vs. PD vs. RA only vs. healthy controls; baseline vs. 3-month follow-up	Prolactin in (GCF) Serum PRL, synovial fluid PRL, DAS28, ESR, periodontal parameters (Probing depth, CAL, PI, GI)	GCF: partial η^2^ = 0.911 (between-group before), 0.755 (between-group after); time effect: 0.789 (RA + PD), 0.712 (chronic periodontitis), Serum: time effect partial η^2^ = 0.292 (RA + PD), 0.119 (chronic periodontitis); between-group partial η^2^ = 0.309 (before SRP), 0.189 (after SRP), SF: partial η^2^ = 0.0003; time effect (d) = 0.834	NR	<0.001 (GCF between groups, before and after); <0.001 (Serum between groups, before and after SRP); time effect: <0.001 (RA + PD), <0.008 (chronic periodontitis); SF: 0.919 (between groups), 0.001 (time effect)	Local GCF and synovial levels of PRL seem to be linked to the disease process of both PD and RA than serum levels. SRP reduced these local levels.
Dolcezza et al. 2024 [60]	Systematic review and meta-analysis	RA + PD	non-surgical periodontal therapy	Non-PD	CAL, DAS28	CAL: −0.56 mm DAS28: −0.39	CAL:−0.82 and −0.31 DAS:−0.46 to −0.31	CAL: 0.001 DAS: <0.001	The present study shows how the control of periodontal disease through non-surgical periodontal treatment can reduce the severity of RA. This finding consistently supports the idea that there is a pathogenic association between these two chronic inflammatory diseases.
Huang et al. 2021 [61]	Meta-analysis (7 RCTs, *n* = 212)	RA + PD vs. non-RA + PD	Scaling and root planing	Scaling and root planning in Non-RA + PD	Probing depth CAL	Probing depth: MD = −0.06 CAL:MD = 0.23	0.18–0.06 −0.01–0.46	0.835 More than 0.05	Anti-TNF-α therapy influenced periodontal microbiota with a higher frequency of T. denticola Methotrexate combined with leflunomide exhibited a higher extension of CAL and anti-TNF-α therapy with methotrexate was associated with a lower extension of CAL (*p* = 0.05). The use of corticosteroids exerted a protective effect on the number of teeth (*p* = 0.027). The type of DMARD affected *P. gingivalis*, T. forsythia and E. nodatum presence. Elevated ACPAs titers were associated with the presence of red complex periodontal pathogens (*p* = 0.025). Bleeding on probing was associated with elevated CPR levels (*p* = 0.05), and ESR was associated with a greater PD (*p* = 0.044) and presence of red complex (*p* = 0.030).

Abbreviations: RA = rheumatoid arthritis; PD = periodontitis; SRP = scaling and root planing; OHI = oral hygiene instructions; DAS28 = Disease Activity Score 28; ESR = erythrocyte sedimentation rate; CRP = C-reactive protein; TNF-α = tumor necrosis factor-alpha; CAL = clinical attachment loss; PPD = probing pocket depth; BOP = bleeding on probing; GI = gingival index; PI = plaque index; SF = synovial fluid; GCF = gingival crevicular fluid; PRL = prolactin; CI = confidence interval; NR = not reported; NS = not significant; DMARD = disease-modifying antirheumatic drug; ACPAs = anti-citrullinated protein antibodies; *P. gingivalis* = *Porphyromonas gingivalis*; *T. denticola* = *Treponema denticola*; *T. forsythia* = *Tannerella forsythia*; *E. nodatum* = *Eubacterium nodatum*.

**Table 4:** Clinical evidence regarding the impact of rheumatoid arthritis treatment on periodontal parameters and inflammatory outcomes.

Study	Design	Population	Intervention	Comparator	Outcomes	Effect Size	95% CI	*p*-Value	Key Finding
Hatipoğlu et al. 2022 [76]	Case-control study	Total *n* = 70 RA with B-cell depletion (rituximab): *n* = 20 RA on DMARDs: *n* = 20 Healthy controls: *n* = 30	B-cell depletion therapy (rituximab ≥ 6 months)	RA patients on conventional DMARD therapy, Non-RA healthy controls	IL-1β MMP-8 Probing depth, CAL, BOP, PI, GI, GCF DAS28, RF, anti-CCP, CRP, ESR	IL-1β (GCF):B-cell depletion: 1.85 ± 1.67 DMARD: 10.50 ± 13.16 Control: 34.12 ± 29.45, MMP-8 (GCF):B-cell depletion: 21.00 ± 4.23 DMARD: 8.16 ± 6.94 Control: 21.45 ± 8.67	NR NR	IL-1β *p* < 0.001 MMP-8 < 0.001	GCF IL-1β levels were significantly lower in B cell depletion group, and MMP-8 levels were significantly lower in DMARD group, suggesting that RA treatments may modify biochemical parameters of GCF. This study suggests that host modulation therapies in RA can reduce local production of IL-1β and MMP-8. Reduction of these inflammatory cytokines and enzymes may have a beneficial effect in controlling periodontal tissue destruction.
Coat et al. 2015 [77]	cross-sectional and longitudinal observational	*n* = 21 RA (2 groups) Group 1: *n* = 11 before rituximab and again 6 months later. Group 2: *n* = 10 two rituximab at the time of periodontal assessment.	Anti-B lymphocyte therapy with Rituximab	RA patients prior to rituximab exposure	Pocket depth and CAL	PD Group 2: 2.06 Group 1: 2.63 CAL Group 2: 2.59 Group 1: 2.9	0.37 0.73 0.8 0.97	*p* < 0.001 *p* < 0.001	Pocket depth and attachment loss were significantly decreased 6 months after treatment with rituximab in group I. Patients from group II had a better periodontal status than patients from group I before treatment with rituximab. Anti-B lymphocyte therapy could be beneficial to PD suggesting a major role of B cells in this disease.
Zhang et al. 2021 [78]	Systematic review and meta-analysis	RA + PD	Anti-rheumatic therapy: DMARDs, anti-TNF-α, anti-IL-6 R agents, anti-B lymphocyte agents, JAK inhibitors	RA + PD without anti-rheumatic agents	Probing depth, CAL, gingival index/modified GI (GI/MGI), BOP, PI	Probing depth: WMD −0.20; CAL: WMD −0.40	Probing depth: −0.33 to −0.07; CAL: −0.66 to −0.15	Probing depth *p* = 0.003; CAL *p* = 0.002	Probing depth, CAL, GI/MGI, and BOP decreased when patients with RA and PD were treated with csDMARDs, anti-B lymphocyte agents, anti-IL-6R agents, or JAK inhibitors. Probing depth and CAL declined after the administration of anti-TNF-α agents.
Kobayashi et al. 2015 [79]	Longitudinal comparative clinical	Total: 60 patients with RA + chronic PD TCZ group: *n* = 20 TNFI group = 40	Tocilizumab (IL-6 receptor inhibitor)	TNFI: infliximab, etanercept, adalimumab, golimumab	GI, BOP, probing depth, CAL	BOP (%) TCZ: Baseline: 8.2 ± 10.2 6 months: 1.9 ± 3.8 TNFI: Baseline: 10.4 ± 11.3 6 months: 6.8 ± 7.6 Probing Depth TCZ: Baseline: 2.57 ± 0.32 6 months: 2.45 ± 0.24 TNFi: Baseline: 2.63 ± 0.31 6 months: 2.51 ± 0.33 CAL TCZ: Baseline: 2.63 ± 0.31 6 months: 2.55 ± 0.31 TNFI: Baseline: 2.72 ± 0.35 6 months: 2.70 ± 0.44	NR	BOP TCZ < 0.017 BOP TNFI < 0.017 Probing depth TCZ < 0.017 Probing depth TNFI < 0.017 CAL TCZ < 0.017 CAL TNFI *p* > 0.017	It may be beneficial effect of TCZ therapy on levels of periodontal inflammation in patients with RA and PD, which might be related to decrease in serum inflammatory mediators.
de Smit et al. 2021 [80]	Longitudinal observational	26 RA patients (14 MTX group, 12 anti-TNF + MTX group)	Methotrexate (MTX) or Anti-TNF-α (etanercept) + MTX	Within-group baseline vs. follow-up	PISA BOP/probing depth/CAL, DAS28, CRP & ESR	NR	NR	PISA: NS (*p* > 0.05) BOP/PD/CAL: NR DAS28: *p* < 0.01 (MTX), *p* < 0.05 (anti-TNF) CRP/ESR: *p* < 0.05	Anti-rheumatic therapy (MTX and anti-TNF) significantly improves RA activity but has no significant effect on periodontal inflammation (PISA, BOP, probing depth.)
Romero-Sanchez et al. 2017 [81]	Cross-sectional observational	179 RA Anti-TNF-α group: *n* = 62 Conventional DMARDs group: *n* = 115	Anti-TNF-α therapy ± methotrexate Conventional DMARDs (including methotrexate ± leflunomide)	Anti-TNF-α vs. DMARDs	CAL, probing depth, BOP plaque index, gingival index, *T. denticola*, *P. gingivalis*, *T. forsythia*, *E. nodatum*, DAS28-ESR, CRP, RF, ACPAs	NR	NR	CAL: *p* = 0.005 (MTX + leflunomide increase), *p* = 0.05 (anti-TNF + MTX decrease) PD: *p* = 0.044 (association with ESR) BOP: *p* = 0.05 (association with CRP) Microbiota: *p* = 0.01 (*T. denticola*), *p* = 0.025 (ACPAs), *p* = 0.030 (ESR) Tooth number (corticosteroids): *p* = 0.027	Anti-TNF-α increase *T. denticola*. Methotrexate combined with leflunomide exhibited a higher extension of CAL, and anti-TNF-α therapy with methotrexate was associated with a lower extension of CAL. The use of corticosteroids protected the number of teeth. The type of DMARD affected *P. gingivalis, T. forsythia* and *E. nodatum* presence. Elevated ACPAs titers were associated with the presence of red complex periodontal pathogens. BOP was associated with elevated CRP levels, and ESR was associated with a greater PD and presence of red complex.

Abbreviations: RA = rheumatoid arthritis; PD = probing depth; *n* = number; CAL = clinical attachment level; BOP = bleeding on probing; GI = gingival index; PI = plaque index; IL-1β = interleukin-1 beta; MMP-8 = matrix metalloproteinase-8; GCF = gingival crevicular fluid; DAS = Disease Activity Score; DMARDs = disease-modifying antirheumatic drugs; csDMARDs = conventional synthetic DMARDs; bDMARDs = biologic DMARDs; JAKi = Janus kinase inhibitors; TNF = tumor necrosis factor; TNFi = tumor necrosis factor inhibitor; MTX = methotrexate; PISA = periodontal inflamed surface area; TCZ = tocilizumab; RF = rheumatoid factor; CRP = C-reactive protein; anti-CCPs = anti-cyclic citrullinated peptides; ACPA = anti-citrullinated protein antibodies; ESR = erythrocyte sedimentation rate; WMD = weighted mean difference; CI = confidence interval; NR = not reported; NS = not significant; *P. gingivalis* = *Porphyromonas gingivalis*; *T. denticola* = *Treponema denticola*; *T. forsythia* = *Tannerella forsythia*; *E. nodatum* = *Eubacterium nodatum*.

## Data Availability

No new data were created or analyzed in this study.
